# miR‐135a‐5p Is a Promising Target to Prevent the Glomerulosclerosis Associated with Podocyte Developmental Toxicity in Offspring Induced by Prenatal Dexamethasone Exposure

**DOI:** 10.1002/advs.202519743

**Published:** 2026-01-30

**Authors:** Xiaoqi Zhao, Haiyun Chen, Yanan Zhu, Zhiping Xia, Hangyuan He, Yutang Liu, Tianshu Yang, Hui Wang, Ying Ao

**Affiliations:** ^1^ Department of Pharmacology Wuhan University School of Basic Medical Sciences Wuhan Hubei China; ^2^ Department of Pharmacy Renmin Hospital of Wuhan University Wuhan Hubei China; ^3^ Department of Reproductive Medicine Center, Xiangyang Central Hospital Affiliated Hospital of Hubei University of Arts and Science Xiangyang Hubei China; ^4^ Division of Joint Surgery and Sports Medicine, Department of Orthopedic Surgery Zhongnan Hospital of Wuhan University Wuhan Hubei China; ^5^ Hubei Provincial Key Laboratory of Developmentally Originated Disease Wuhan Hubei China

**Keywords:** developmental toxicity, epigenetic, miRNA therapy, podocyte, prenatal dexamethasone exposure, renal disease

## Abstract

Podocyte developmental defects are pivotal in initiating glomerulosclerosis. Although antenatal glucocorticoid therapy improves neonatal outcomes, prenatal dexamethasone exposure (PDE) may trigger intrauterine programming that predisposes offspring to chronic kidney disease, yet its mechanisms remain elusive. Here, we show that PDE disrupts podocyte differentiation and induces glomerulosclerosis in adult rat offspring. Mechanistically, PDE downregulated KLF4 and upregulated miR‐135a‐5p, which targets KLF4. In differentiating metanephric mesenchymal stem cells, dexamethasone reduced KLF4 while increasing miR‐135a‐5p expression. Chromatin analyses revealed that activated glucocorticoid receptor (GR) bound the miR‐135a‐5p promoter and recruited P300, enhancing histone acetylation and supporting sustained upregulation of miR‐135a‐5p. Elevated miR‐135a‐5p suppressed KLF4, impairing podocyte development and promoting long‐term glomerular injury. Early administration of a miR‐135a‐5p antagomir partially restored podocyte markers and ameliorated PDE‐induced sclerosis. These findings identify a previously unrecognized GR–P300–miR‐135a‐5p/KLF4 epigenetic axis governing fetal programming of podocyte injury. Our work provides mechanistic insight into the developmental origins of glucocorticoid‐induced kidney disease and highlights miR‐135a‐5p as a promising biomarker and potential therapeutic target for preventing fetal‐origin glomerulosclerosis.

## Graphical abstract

Prenatal dexamethasone exposure (PDE) programs persistent podocyte developmental injury and adult glomerulosclerosis. Mechanistically, glucocorticoid receptor (GR) binds the miR‐135a‐5p promoter and recruits the histone acetyltransferase p300, increasing promoter histone acetylation and sustaining miR‐135a‐5p expression. Elevated miR‐135a‐5p suppresses Kruppel‐like factor 4 (KLF4), impairing podocyte differentiation; early miR‐135a‐5p antagonism partially mitigates pathology

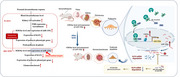

## 1 Introduction

Dexamethasone is a common synthetic glucocorticoid widely used in clinical medicine and animal husbandry due to its powerful anti‐inflammatory and anti‐allergic properties [1, 2, 3]. In addition, dexamethasone is widely used in preterm pregnant women to promote fetal lung maturation and reduce neonatal mortality [4]. It is also commonly used in the management of pregnancy complications that pose a persistent risk of preterm birth, such as multiple pregnancies and placenta previa hemorrhage. A 2014 survey by the World Health Organization (WHO) found that the average rate of antenatal glucocorticoid therapy for pregnant woman in all countries was 54%, with the highest rate reaching 91% [5]. Furthermore, the COVID‐19 pandemic has led to more people being treated with dexamethasone in recent years [6]. However, the health effects of maternal dexamethasone exposure during pregnancy on the offspring remain a concern [7]. Studies have found that maternal dexamethasone exposure during pregnancy can increase disease susceptibility and even lead to disease in the offspring [8, 9, 10, 11, 12]. There is still no effective breakthrough in the study of the occurrence mechanism of this fetal‐derived health hazard and the specific preventive and curative measures.

Podocytes are glomerular epithelial cells that are tightly bound to the basement membrane through a unique foot processes and thus form a slit diaphragm that regulates the selective filtration of substances between blood and urine. Podocytes provide mechanical support and prevent leakage of proteins and macromolecules, and are therefore critical for maintaining the integrity of the glomerular filtration barrier. Injury or apoptosis of podocytes leads to dysfunction of the filtration barrier and is an important pathogenic factor in many renal diseases. Glomerulosclerosis is a clinicopathological syndrome characterized by glomerular glassy degeneration, with proteinuria and progressive deterioration of renal function as the main clinical manifestations. Whereas podocytes are terminally differentiated cells, they are not regenerative after differentiation and maturation, and their number depends largely on their embryonic development [13, 14]. Epidemiologic data show that low birth weight individuals have significantly reduced renal podocyte numbers and are at increased risk for glomerular disease in adulthood [15, 16, 17]. Notably, preliminary evidence suggests that prenatal glucocorticoid exposure may impact kidney development by altering epigenetic programming [18, 19, 20], although its specific role in podocyte lineage specification remains unclear. In our previous study, we found that PDE could cause glomerulosclerosis in adult rat offspring [21], but the mechanism has not been elucidated. It has been confirmed that podocyte injury is the initiating factor leading to glomerulosclerosis [13, 22, 23, 24]. Therefore, we hypothesized that PDE‐induced glomerulosclerosis may be related to podocyte developmental toxicity.

MicroRNAs (miRNAs) are a class of small non‐coding RNAs widely found in organisms that negatively regulate target gene expression at the post‐transcriptional level, mainly by targeting messenger RNAs (mRNAs) [25]. Recent studies have shown that miRNAs play a key role in regulating the development and function of a variety of tissues and organs, including the kidney [26]. Increasing evidence suggests that miRNAs may mediate podocyte damage by regulating genes associated with cell differentiation, proliferation, apoptosis, and cytoskeleton dynamics [27]. However, their roles and potential mechanisms in podocyte development have not been elucidated in detail. An in‐depth understanding of the roles played by specific miRNAs in podocyte development and their response to toxic exposures may reveal novel mechanisms of renal developmental toxicity and could provide potential therapeutic targets for the prevention or mitigation of renal disease.

In this study, we delved into the mechanism of intrauterine programming of PDE‐induced glomerulosclerosis in offspring in the perspective that dexamethasone‐induced miRNA epigenetic alterations mediating the developmental toxicity of podocytes. In addition, we investigated the prevention and treatment of fetal‐derived glomerulosclerosis associated with PDE‐induced podocyte developmental toxicity by miRNA intervention on PDE offspring. This study can provide theoretical and experimental evidence to reveal the mechanism of fetal‐derived renal disease and to explore the effective prevention and treatment strategy of dexamethasone developmental nephrotoxicity.

## 2 Results

### 2.1 PDE Causes Developmental Toxicity of Renal Podocytes in Offspring Rats

**FIGURE 1:**
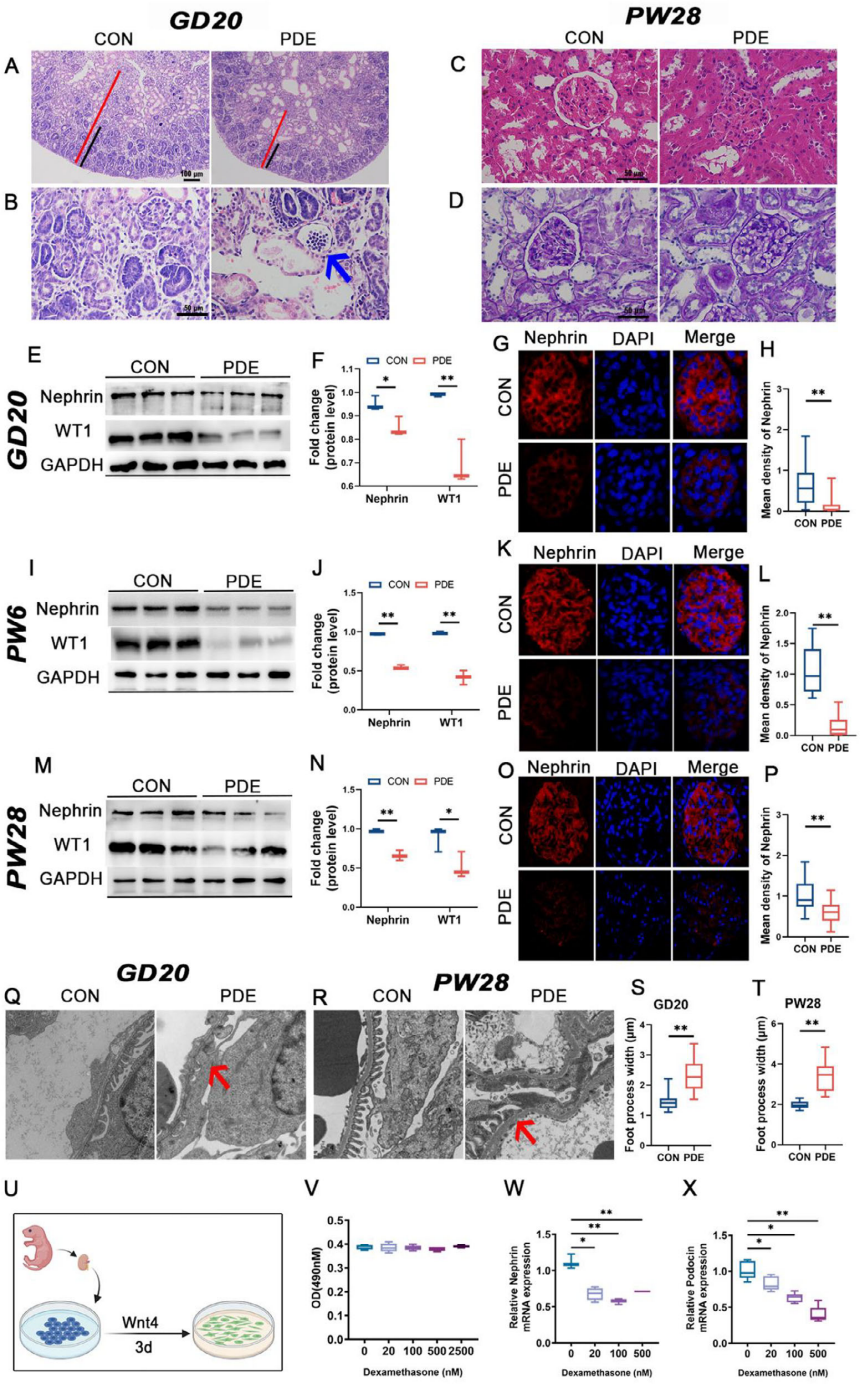
The effect of prenatal dexamethasone exposure on renal structure and function of the male offspring rats. (A) Fetal kidney sections were stained with hematoxylin and eosin (HE) reagents (magnification ×100). The red and black lines represent cortical and nephrogenic zones, respectively. (B) Fetal kidney sections were stained with HE (magnification ×400). The blue arrow indicates dysplasia of the glomerulus. (C) Adult kidney sections were stained with HE (magnification ×400). (D) Adult kidney sections were stained with Periodic acid‐Schiff's reagents (PAS) (magnification ×400). (E, F) Protein expression of Nephrin, WT1 in fetal kidney, *n* = 4. (G, H) Immunofluorescent staining of Nephrin in fatal kidney (magnification: ×400), *n* = 4. (I, J, M, N) Protein expression of Nephrin, WT1 in the offspring kidneys at PW6 and PW28, *n* = 3. (K, L, O, P) Immunofluorescent staining of Nephrin in the offspring kidneys at PW6 and PW28 (magnification: ×400), *n* = 4. (Q, R) Morphology of podocytes in the fetal kidney and the adult kidney was observed by transmission electron microscopy (TEM) (magnification: ×10000). Red arrows indicate podocyte foot process effacements. (S, T) Representative TEM images and quantifications of foot process width. (U) Schematic diagram of cell processing. (V) MTS experiment in the differentiation cells treated with dexamethasone, *n* = 6. (W, X) mRNA expressions of Nephrin and Podocin in the differentiation cells treated with dexamethasone, *n* = 6. Each fetal kidney sample was pooled by three or six fetal offspring kidneys from two pregnant rats. Each adult kidney sample was pooled by two offspring kidneys from two pregnant rats. Two‐group comparisons were conducted using Student's *t*‐test or Mann–Whitney U test; for multiple‐group comparisons, one‐way ANOVA was applied, followed by post hoc Dunnett's test. Mean ± S.E.M. ^*^ *p* < 0.05, ^**^ *p* < 0.01 vs control. GD, gestational day; PW, postnatal week; CON, control; PDE, prenatal dexamethasone exposure; WT1, Wilms’ tumor 1; GAPDH, glyceraldehyde phosphate dehydrogenase.

In order to investigate the effect of PDE on the development of podocytes in offspring kidneys, we examined the renal morphology and podocyte development indices of offspring before birth (gestational day 20, GD20) and at different time points after birth (postnatal week 6 and 28, PW6 and 28). Morphological results showed that the PDE group displayed fetal kidney dysplasia compared to the control group, as exhibited by significant thinning of the cortical and nephrogenic zones, a distinct reduction in the number of mature glomeruli (Figure 1A), emptying of glomerular Bowman's capsule, and poorly developed capillary network (Figure 1B). At PW28, the PDE group showed glomerular hyperplasia (Figure 1C), basement membrane thickening, narrowing of the lumen of Bowman's space, and focal glomerulosclerosis (Figure 1D). The above results were consistent with the results of previous studies [21], suggesting that PDE leads to fetal renal dysplasia and glomerulosclerosis in adulthood. Further, we examined relevant indicators of podocyte morphology and development. Western blot and immunofluorescence results showed that the protein expression of podocyte functional genes Nephrin and WT1 was significantly reduced in the PDE group at GD20 (prenatal), PW6 (puberty), and PW28 (adulthood) (Figure 1E–P, *p* < 0.05 or 0.01). Under electron microscopy, the kidneys in the PDE group at both GD20 and PW28 showed thickening of the glomerular basement membrane, large fusion or effacement of foot processes, and a significant reduction in the number of foot processes (Figure 1Q,R). The above results suggested that PDE resulted in morphological and functional dysplasia of renal podocytes in the offspring, which persisted until the offspring reached adulthood.

Dexamethasone can cross the placenta into the fetus. Thus, we hypothesized that PDE‐induced podocyte developmental toxicity of offspring may be related to the direct toxicity of dexamethasone. Therefore, we constructed an in vitro podocyte differentiation model and used this cellular model to explore the effects of dexamethasone on podocyte differentiation. Wnt4 (100 ng/mL) was administered for 3 d to induce the directional differentiation of primary metanephric mesenchymal stem cells (MMSCs) toward podocytes (Figure 1S). Flow cytometry assay showed a positive rate of 96.7% for differentiated podocytes (Figure S1A). Meanwhile, the mRNA expression of both Nephrin and Podocin was significantly elevated compared with undifferentiated MMSCs (Figures S1B,C and S2, *p* < 0.01), suggesting that the model of directional differentiation of podocytes was successfully established. Subsequently, based on the model of podocyte differentiation, cells were treated with different concentrations of dexamethasone at the same time. MTS results showed that cell viability was not affected by dexamethasone treatment for 3 d in the concentration range of 0–2500 nM (Figure 1T, *p* > 0.05). And the Reverse transcription and quantitative PCR (RT‐qPCR) results revealed that dexamethasone significantly decreased the mRNA levels of Nephrin and Podocin (Figure 1U,V, *p* < 0.05 or 0.01). The above results suggested that dexamethasone can directly inhibit podocyte differentiation, which leads to developmental toxicity of offspring podocytes.

### 2.2 Altered miR‐135a‐5p/ Kruppel‐Like Factor 4 (KLF4) Expression Mediates PDE‐Induced Impaired Podocyte Development in Offspring Rats

**FIGURE 2:**
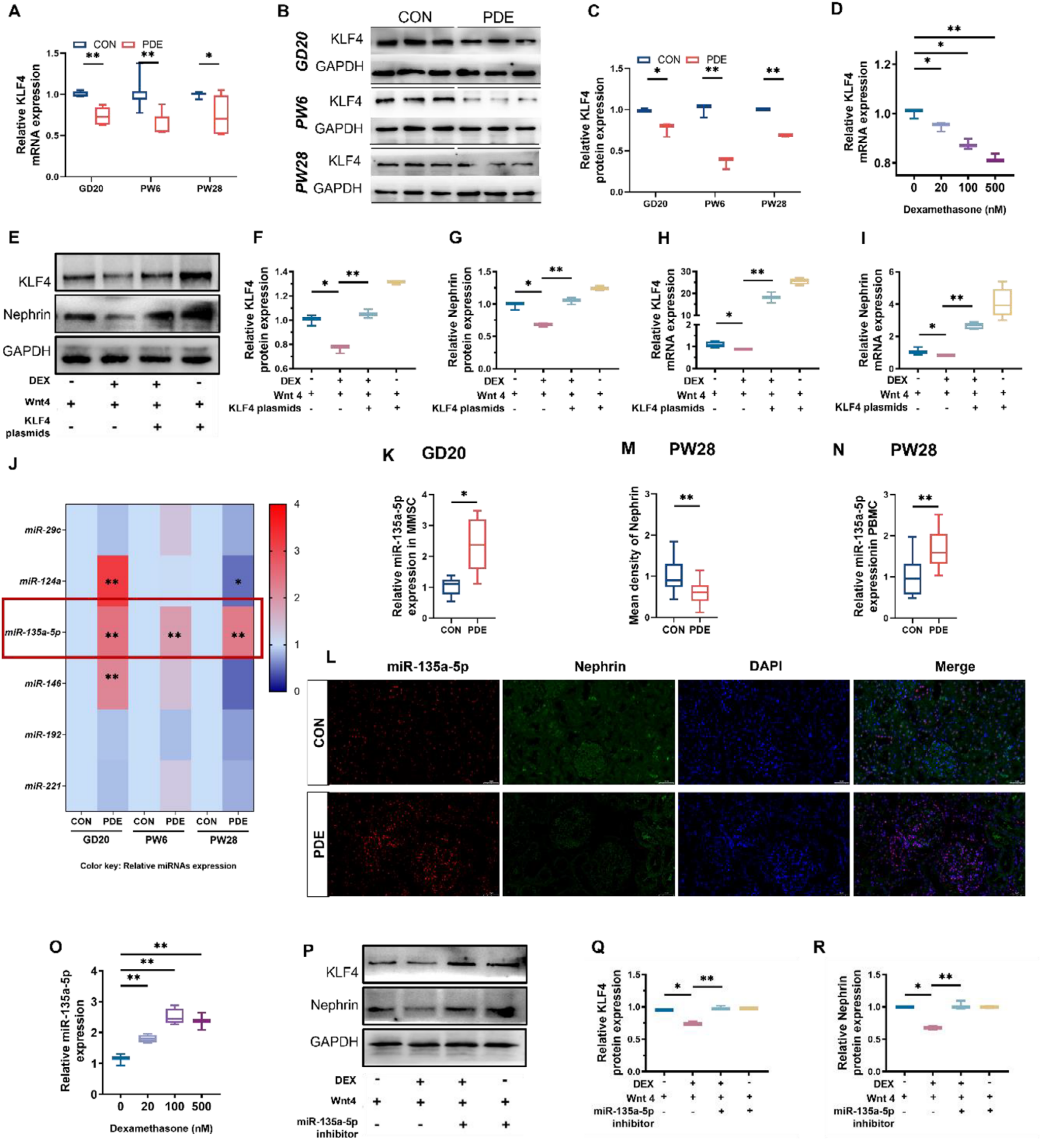
Effects of PDE on renal miR‐135a‐5p and KLF4 expression. (A) mRNA expression of KLF4 in the offspring kidneys at GD20, PW6, and PW28, *n* = 6. (B, C) Protein abundance of KLF4 in the offspring kidneys at GD20, PW6, and PW28, *n* = 4. (D) KLF4 mRNA expression in the differentiation cells treated with dexamethasone, *n* = 6. (E–G) Protein abundance of KLF4 and Nephrin was detected by western blot in the differentiation cells with 500 nM dexamethasone in combination with KLF4 plasmids, *n* = 3. (H, I) mRNA expression of KLF4 and Nephrin was determined by RT‐qPCR in the differentiation cells with 500 nM dexamethasone in combination with KLF4 plasmids, *n* = 6. (J) miRNAs expression in the kidney, *n* = 3. (K) miR‐135a‐5p expression in MMSCs of offspring rats, *n* = 6. (L, M) In situ hybridization of miR‐135a‐5p and co‐staining with Nephrin immunofluorescence in the kidneys of adult offspring rats. *n* = 3. (N) miR‐135a‐5p expression in PBMC of male offspring at PW28. *n* = 8∼11. (O) miR‐135a‐5p expression in the differentiation cells treated with dexamethasone, *n* = 6. (P–R) Protein abundance of KLF4 and Nephrin was detected by western blot in the differentiation cells with 500 nM dexamethasone in combination with miR‐135a‐5p inhibitor plasmids, *n* = 3. Each fetal kidney sample was pooled by three or six fetal offspring kidneys from two pregnant rats. Each adult kidney sample was pooled by two offspring kidneys from two pregnant rats. The *p*‐value was calculated using Student's *t*‐test or One‐way ANOVA with the post hoc Dunnett's *t*‐test or Tukey's post hoc test, mean ± S.E.M. ^*^ *p <* 0.05, ^**^ *p <* 0.01 *vs* control. GD, gestational day; PW, postnatal week; CON, control; PDE, prenatal dexamethasone exposure; KLF4, Krüppel‐like factor4; GAPDH, glyceraldehyde phosphate dehydrogenase; DEX: dexamethasone.

KLF4 is mainly expressed in epithelial cell‐containing tissues, and has been found to be abundantly expressed in both fetal and adult kidneys [28, 29]. KLF4 promotes podocyte development and maintains the podocyte phenotype by upregulating the expression of Nephrin, WT1, and Podocin [28]. To explore the mechanism that PDE inhibits phenotypic gene expression in offspring renal podocytes, we examined the expression of KLF4 in offspring kidneys at different time points from intrauterine to postnatal. The results showed that the mRNA and protein expression of KLF4 of offspring kidneys in the PDE group was significantly lower than that in the control group at GD20, PW6, and PW28 (Figure 2A–C, *p* < 0.05 or 0.01), suggesting that PDE may suppress the podocyte phenotype by inhibiting the expression of KLF4 in the offspring kidneys. At the cellular level, we found that dexamethasone significantly reduced KLF4 mRNA and protein expression in the podocyte differentiation model (Figure 2D–F, *p* < 0.05 or 0.01). Further, we utilized a KLF4 overexpression plasmid to transfect MMSCs and found that the inhibitory effect of dexamethasone on the mRNA and protein expression of Nephrin was reversed by KLF4 overexpression (Figure 2E–I, *p* < 0.05 or 0.01). The above results suggested that dexamethasone may mediate podocyte developmental toxicity by inhibiting KLF4.

It has been reported that KLF4 is closely related to miRNAs and its expression is susceptible to miRNA regulation [30, 31]. To explore the mechanism of inhibition of KLF4 by PDE, we screened a series of miRNAs that might target KLF4 based on literature and Targetscan database prediction, and detected them in the fetal and adult offspring kidneys [32, 33]. The results showed that only the expression of miR‐135a‐5p in the PDE group was significantly higher than the control group at all the time points of GD20, PW6, and PW28 (Figure 2J, *p* < 0.01). MMSCs were extracted from the fetal kidneys of the offspring rats for detection. It was found that compared with the control group, the expression of miR‐135a‐5p in MMSCs of the PDE offspring fetal mice (GD20) increased (Figure 2K, *p* < 0.01). In the in situ hybridization experiment results of miR‐135a‐5p in adult offspring kidneys, although we found that miR‐135a‐5p was not only expressed in podocytes, through co‐staining of miR‐135a‐5p with the podocyte marker Nephrin, it was discovered that the miR‐135a‐5p signal highly overlaps with the podocyte region within the glomerulus. Moreover, the expression of miR‐135a‐5p in adult progeny podocytes of PDE was higher than that in the control group (Figure 2L,M, *p* < 0.01). This result suggested that miR‐135a‐5p may be closely related to PDE‐induced developmental toxicity in offspring renal podocytes. Interestingly, we also found that the expression of miR‐135a‐5p in PBMC of adult male offspring in PDE group (PW28) was higher than that in the control group (Figure 2N, *p* < 0.01). In vitro experiments showed that dexamethasone treatment significantly elevated miR‐135a‐5p expression in MMSCs (Figure 2O, *p* < 0.05 or 0.01). Besides, the inhibitory effect of dexamethasone on the protein expression of KLF4 and Nephrin was reversed after transfection of cells with miR‐135a‐5p inhibitor plasmid (Figure 2P–R, *p* < 0.05 or 0.01). Taken together, this suggested that dexamethasone inhibits KLF4 expression through upregulation of miR‐135a‐5p, thus inhibiting podocyte differentiation and development.

### 2.3 High Histone Acetylation/Expression of miR‐135a‐5p is Involved in Mediating Developmental Toxicity in PDE Offspring Rat Podocytes

**FIGURE 3:**
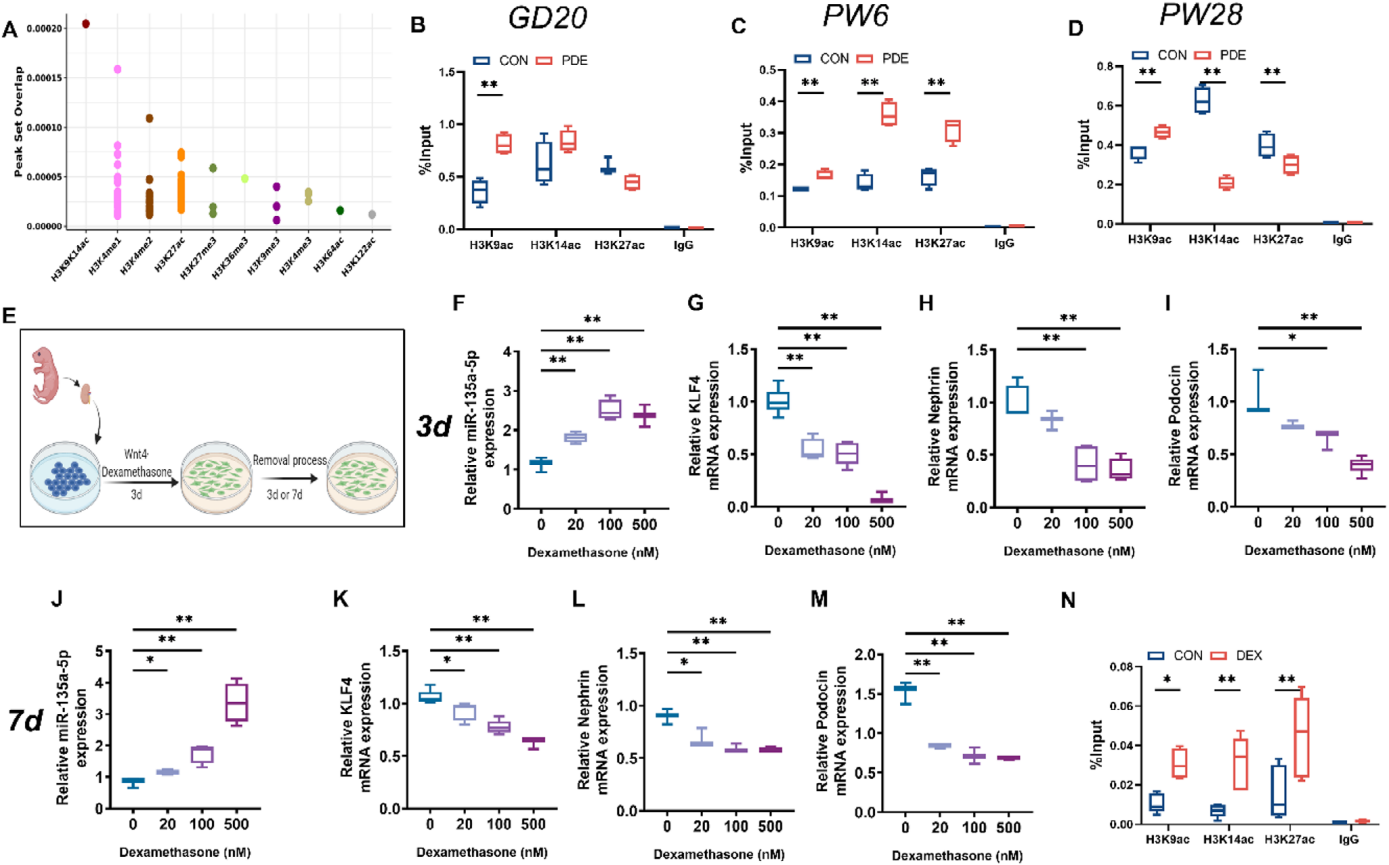
Effects of PDE on the epigenetic programming of miR‐135a‐5p in the kidneys of offspring. (A) Predicted histone epigenetic forms of miR‐135a‐5p. (B–D) Enrichment of H3K9ac, H3K14ac, and H3K27ac in the miR‐135a‐5p promoter was analyzed using ChIP‐PCR in the offspring kidneys from GD20 to PW28, *n* = 4. (E) Schematic diagram of cell processing. (F–I) mRNA expression of miR‐135a‐5p, KLF4, Nephrin, and Podocin was determined by RT‐qPCR 3 days after the removal of dexamethasone, *n* = 6. (J–M) mRNA expression of miR‐135a‐5p, KLF4, Nephrin, and Podocin was determined by RT‐qPCR 7 days after the removal of dexamethasone, *n* = 6. (N) Enrichment of H3K9ac, H3K14ac, and H3K27ac in the miR‐135a‐5p promoter was analyzed through ChIP‐PCR in the primary MMSCs treated with 500 nM dexamethasone, *n* = 3. Each fetal kidney sample was pooled by three or six fetal offspring kidneys from two pregnant rats. Each adult kidney sample was pooled by two offspring kidneys from two pregnant rats. The *p*‐value was calculated using Student's *t*‐test or One‐way ANOVA with the post hoc Tukey's post hoc test, mean ± S.E.M. ^*^ *p <* 0.05, ^**^ *p <* 0.01 *vs* control. GD, gestational day; PW, postnatal week; CON, control; PDE, prenatal dexamethasone exposure; KLF4, Krüppel‐like factor 4; GAPDH, glyceraldehyde phosphate dehydrogenase.

Epigenetic modification is a key mechanism of intrauterine programming, with histone modification being a common form of such modification. The high expression of miR‐135a‐5p persists from prenatal to adult offspring, suggesting that the mechanism may be related to epigenetic regulation. The most likely epigenetic form of histone present in the miR‐135a‐5p promoter region as predicted by the Cistrome Data Browser, is histone acetylation (Figure 3A). Therefore, we examined histone acetylation levels and methylation levels in the miR‐135a‐5p promoter region of offspring kidneys at different time points. The results showed that at GD20, PW6, and PW28, the levels of H3K9ac in the renal miR‐135a‐5p promoter region of the offspring in the PDE group were significantly elevated compared with the control group (Figure 3B–D; Figure S2A,B, *p* < 0.01), indicating that the high levels of H3K9ac were carried over from intrauterine to postnatal period. These findings support that the mechanism of PDE‐induced altered programming of high miR‐135a‐5p expression in the offspring kidney may be related to the elevated level of H3K9ac in the miR‐135a‐5p promoter region.

Next, we explored at the cellular level whether dexamethasone could directly induce programming changes of miR‐135a‐5p. Cells were treated with dexamethasone for 3 d in the podocyte differentiation model and continued to culture the cells for 3 or 7 days after dexamethasone was withdrawn (Figure 3E). The results showed that compared with the control group, the expression of miR‐135a‐5p was still significantly higher 3 and 7 days after the withdrawal of dexamethasone, and the downstream gene expression of KLF4, Nephrin, and Podocin was significantly lower (Figure 3F–M, *p* < 0.05 or 0.01). The above results suggested that dexamethasone has a durable effect on the upregulation of miR‐135a‐5p expression, consistent with in vivo results. Further, to verify the involvement of the epigenetic pathway in mediating the programming of high miR‐135a‐5p expression, we detected the histone acetylation level of miR‐135a‐5p promoter after treating MMSCs with dexamethasone. The results revealed that dexamethasone significantly upregulated the H3K9ac, H3K14ac, and H3K27ac levels of miR‐135a‐5p (Figure 3N, *p* < 0.05 or 0.01). Taken together, we speculated that dexamethasone mediates podocyte developmental toxicity by up‐regulating the histone acetylation level of miR‐135a‐5p, leading to miR‐135a‐5p high expression programming.

### 2.4 Glucocorticoid Receptor (GR)/P300 Mediates miR‐135a‐5p Histone Hyperacetylation in PDE Offspring Rats

**FIGURE 4:**
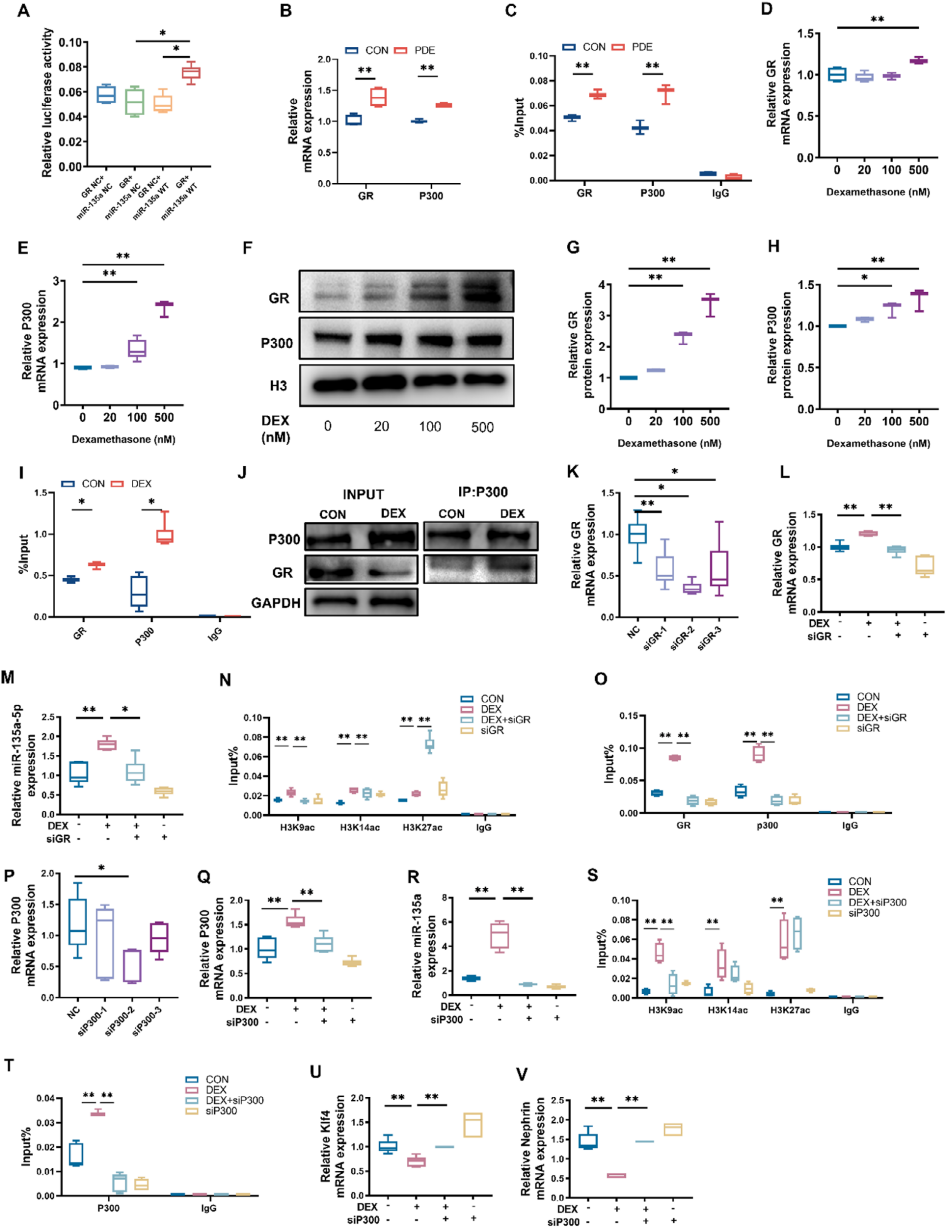
Effects of PDE on the GR/P300 in the fetal kidneys of offspring. (A) GR and miR‐135a‐5p promoter luciferase reporter gene system. (B) mRNA expressions of GR and P300 in the fetal kidney were determined by RT‐qPCR. (C) Enrichment of GR and P300 in the miR‐135a‐5p promoter was analyzed through ChIP‐PCR in the fetal kidney. (D, E) The mRNA expression of GR and P300 was determined by RT‐qPCR in the primary MMSCs treated with dexamethasone for 24 h, *n* = 6. (F–H) Protein expressions of GR and P300 were determined by western blot in the primary MMSCs treated with dexamethasone for 24 h, *n* = 3. (I) Enrichment of GR and P300 in the miR‐135a‐5p promoter was analyzed through ChIP‐PCR in the primary MMSCs treated with 500 nM dexamethasone 24 h. (J) Analysis of the interaction between GR and P300 by Co‐IP. (K) Knockdown of GR expression using three siRNAs, *n* = 3. (L, M) The mRNA expression of GR and miR‐135a‐5p in MMSCs treated with dexamethasone in combination with siGR was determined by RT‐qPCR, *n* = 6. (N) Enrichment of H3K9ac, H3K14ac, and H3K27ac in the miR‐135a‐5p promoter was analyzed through ChIP‐PCR in MMSCs treated with dexamethasone in combination with siGR, *n* = 3. (O) Enrichment of GR and P300 in the miR‐135a‐5p promoter was analyzed through ChIP‐PCR in MMSCs treated with dexamethasone in combination with siGR, *n* = 3. (P) Knockdown of P300 expression using three siRNAs, *n* = 3. (Q, R) The mRNA expression of P300 and miR‐135a‐5p in MMSCs treated with dexamethasone in combination with siP300 was determined by RT‐qPCR, *n* = 6. (S) Enrichment of H3K9ac, H3K14ac, and H3K27ac in the miR‐135a‐5p promoter was analyzed through ChIP‐PCR in MMSCs treated with dexamethasone in combination with siP300, *n* = 3. (T) Enrichment of P300 in the miR‐135a‐5p promoter was analyzed through ChIP‐PCR in MMSCs treated with dexamethasone in combination with siP300, *n* = 3. (U, V) The mRNA expression of KLF4 and Nephrin in MMSCs treated with dexamethasone in combination with siP300 was determined by RT‐qPCR, *n* = 6. Each fetal kidney sample was pooled by three or six fetal offspring kidneys from two pregnant rats. The *p*‐value was calculated using Student's *t*‐test or One‐way ANOVA with Dunnett's *t*‐test or Tukey's *t*‐tests, mean ± S.E.M. ^*^ *p <* 0.05, ^**^ *p <* 0.01 *vs* control. GR, Glucocorticoid receptor; WT, Wild‐Type; CON, control; PDE, prenatal dexamethasone exposure; H3, histone H3; DEX: dexamethasone.

Dexamethasone regulates target gene expression mainly by binding to GR, promoting the entry of GR into the nucleus, and binding to the glucocorticoid responsive elements (GRE) of target genes [34]. The presence of GRE in the miR‐135a‐5p promoter region was predicted by JASPAR software. To validate this predicted binding, we established a miR‐135a‐5p promoter luciferase reporter gene system using HEK293T cells. It was found that luciferase activity was enhanced after co‐transfection of GR plasmid and miR‐135a‐5p promoter sequence (Figure 4A, *p* < 0.05), suggesting the presence of GRE in the miR‐135a‐5p promoter region.

Studies have suggested that GR recruits the transcriptional coactivator P300 to the promoter region of target genes and promotes gene transcription by loosening the superhelical structure of chromatin through histone acetylation modification [35]. In order to explore the molecular mechanism that PDE up‐regulates the level of miR‐135a‐5p histone acetylation, we examined the mRNA expression of GR and P300 in fetal kidneys and found that they were significantly up‐regulated in the PDE group compared with the control group (Figure 4B, *p* < 0.05 or 0.01). Besides, the enrichment of GR and P300 at the miR‐135a‐5p promoter region was significantly enhanced (Figure 3C–E, *p* < 0.01). In vitro, we found that the treatment of rat MMSCs with different concentrations of dexamethasone resulted in a significant increase in mRNA expression and intranuclear protein expression of GR and P300 (Figure 4F–H, *p* < 0.05 or 0.01). ChIP‐qPCR results showed that the enrichment of GR and P300 at the miR‐135a‐5p promoter region was significantly increased in dexamethasone‐treated MMSCs (Figure 4I, *p* < 0.05 or 0.01). Co‐IP assay results also showed that the binding of P300 to GR was enhanced in the dexamethasone‐treated cells compared with the control group (Figure 4J). Further, we knocked down the expression of GR or P300 in MMSCs using siRNA, and found that the knockdown of GR or P300 reversed the increase of H3K9ac levels, enrichment of GR or P300 to the miR‐135a‐5p promoter region, and the expression of miR‐135a‐5p expression (Figure 4K,L,P,Q, *p* < 0.05 or 0.01) (Figure 4M,N,R,S, *p* < 0.05 or 0.01), and the decrease of KLF4 and Nephrin expression (Figure 4U,V, *p* < 0.01) induced by dexamethasone. (Figure 4O,T, *p* < 0.01). These results suggested that dexamethasone may promote GR to bind the miR‐135a‐5p promoter and recruit P300, increasing promoter acetylation and miR‐135a‐5p transcription.

### 2.5 In *vivo* Intervention with miR‐135a‐5p Reverses Renal Podocyte Damage and Glomerulosclerosis in PDE Offspring

**FIGURE 5:**
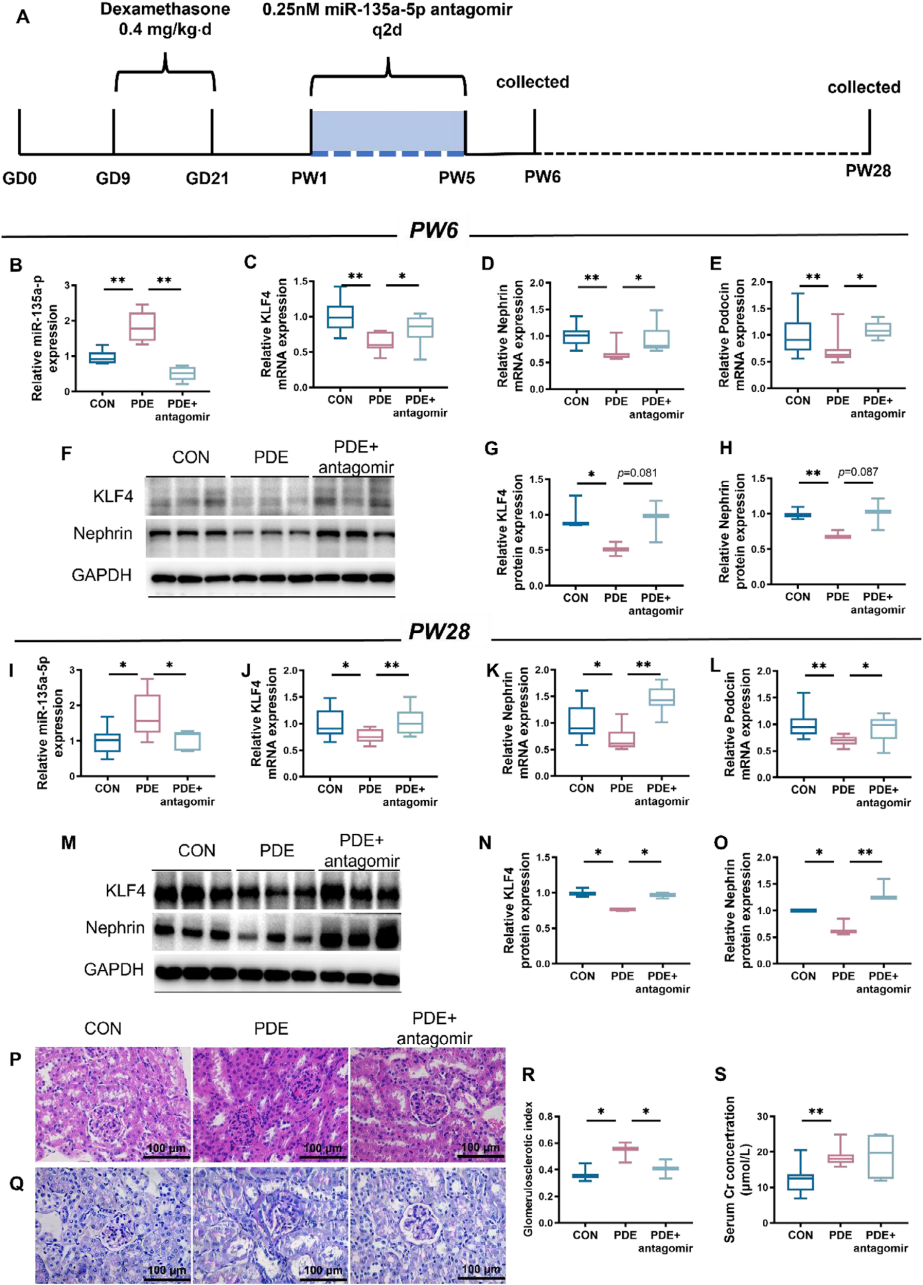
Effects of miR‐135a‐5p antagomir on the mice kidney of PDE offspring. (A) The schematic illustration of the miR‐135a‐5p intervention experiment. (B–E, I–L) The mRNA expression of miR‐135a‐5p, KLF4, Nephrin, and Podocin in the offspring kidneys at PW6 or PW28. *n* = 8. (F–H, M–O) The protein expression of KLF4 and Nephrin in the offspring kidneys at PW6 or PW28. *n* = 3. (P) Kidney sections were stained with hematoxylin and eosin reagents (magnification ×400). *n* = 3. (Q) Kidney sections were stained with Periodic acid‐Schiff's reagents (magnification ×400). *n* = 3. (R) Glomerulosclerotic index. *n* = 3. (S) Serum creatinine level of adult offspring mice. *n* = 8. The *p*‐value was calculated using One‐way ANOVA with Tukey's post hoc test, mean ± S.E.M. ^*^ *p* < 0.05, ^**^ *p* < 0.01 vs control. PW, postnatal week; CON, control; PDE, prenatal dexamethasone exposure; KLF4, Krüppel‐like factor 4; GAPDH, glyceraldehyde phosphate dehydrogenase.

Finally, to investigate whether miR‐135a‐5p could be a target for early intervention in PDE‐induced podocyte developmental toxicity, we administered miR‐135a‐5p antagomir to male PDE offspring mice from PW1 until PW5 (Figure 5A). Morphological and podocyte‐related indices were detected at PW6 and PW28. We found that miR‐135a‐5p antagomir reversed the elevation of miR‐135a‐5p expression and PDE‐induced downregulation of KLF4, Nephrin, and Podocin at both PW6 and PW28 (Figure 5B–E,I–L, *p* < 0.05 or 0.01), and increased the protein expression of KLF4 and Nephrin (Figure 5F–H,M–O). Administration of miR‐135a‐5p antagomir also alleviated PDE‐induced glomerular hyperplasia and sclerosis and reduced the glomerulosclerosis index (GSI) at PW28 (Figure 5P–R, *p* < 0.05). While there was no significant reversal effect on serum creatinine levels (Figure 5S). Taken together, these data indicated that early life intervention with miR‐135a‐5p can partially reverse PDE‐induced renal podocyte damage and glomerulosclerosis in adult offspring.

## 3 Discussion

In this study, we investigated the effects of PDE on renal podocyte development and renal function in offspring rats and explored possible prevention and intervention targets for fetal‐derived glomerulosclerosis. Specifically, we observed that renal podocyte dysplasia in PDE offspring, which persists from intrauterine to postnatal life, may have contributed to glomerulosclerosis. The epigenetic alterations and expression of miR‐135a‐5p may be involved in mediating the impaired podocyte development induced by PDE. And the intervention of miR‐135a‐5p early in life partially reversed the PDE‐induced podocyte impairment and glomerulosclerosis phenotype in adult offspring. In a word, these findings revealed previously unknown cellular and molecular mechanisms for the development of fetal‐derived glomerulosclerosis induced by PDE, and provided new perspectives to revisit the current pros and cons of dexamethasone use during pregnancy. Meanwhile, our study provides promising miRNA strategies for the prevention and treatment of fetal‐derived renal diseases.

Podocytes are glomerular epithelial cells that play a key role in maintaining glomerular filtration function. The injury or loss of podocytes leads to the onset and progression of glomerulosclerosis [13, 23]. During differentiation, podocytes develop regularly spaced foot processes that control glomerular filtration through the slit diaphragms. The podocyte‐specific marker genes Nephrin and Podocin are important for maintaining the structural and functional integrity of podocytes [36]. Nephrin is a transmembrane protein that is an essential component of the podocyte slit diaphragm. Podocin is another key protein in the slit diaphragm that works closely with Nephrin to maintain the structure and function of the glomerular filtration barrier. In the embryonic period, the absence of the podocyte marker genes is a sign of abnormal podocyte development [37]. In adulthood, their reduced expression leads to disruption of podocyte structure and filtration function [38]. Since podocytes are terminally differentiated cells, the accepted consensus is that the final number of podocytes is determined at the end of kidney development [39]. Understanding the impact of abnormal podocyte development in early life on the pathogenesis of renal disease may be important for the development of new pharmacologic interventions for the treatment of fetal‐derived renal disease. KLF4, a key transcription factor upstream of Nephrin and Podocin, has been reported to promote podocyte differentiation [29] and is required to maintain podocyte stability [40]. In proteinuric animals and humans, reduced KLF4 expression leads to proteinuria. Adriamycin‐induced proteinuria was found to be significantly exacerbated in podocyte‐specific KLF4 KO mice. In addition, restoration of KLF4 expression by tail vein injection or podocyte‐specific transgenesis in diseased glomeruli induced restoration of the podocyte marker Nephrin with a concomitant reduction in albuminuria [28]. Our previous study found that caffeine exposure during pregnancy resulted in decreased KLF4 expression in the kidneys of offspring rats and may be associated with the development of glomerulosclerosis in adult offspring [41]. This suggests that aberrant KLF4 expression in early life and its effect on podocyte differentiation may play an important role in the progression of fetal‐derived kidney disease. In this study, we found that the expression of podocyte marker genes and KLF4 was reduced in fetal and adult kidneys of PDE offspring, accompanied by podocyte ultrastructural abnormalities. This suggested that PDE can cause developmental toxicity of podocytes. Meanwhile, consistent with the results of a previous animal study [21], we observed the development of glomerulosclerosis in adult PDE offspring. Decreased numbers of podocytes are thought to be one of the initiators of focal and segmental glomerulosclerosis (FSGS) [24], and we therefore hypothesized that podocyte developmental toxicity may be involved in mediating fetal‐derived glomerulosclerosis induced by PDE. Besides, in an in vitro model of MMSCs differentiation to podocytes, dexamethasone reduced KLF4 and Nephrin expression, whereas overexpression of KLF4 reversed the inhibition of Nephrin expression by dexamethasone. Our study demonstrated that KLF4 plays a crucial role in PDE‐induced developmental toxicity in podocytes.

Studies in recent years have shown that miRNAs have become emerging therapeutic targets for various diseases, due to their extensive involvement in the genesis and development of various diseases. Abnormal expression of miRNAs has been reported to affect fetal kidney development and physiological functions of the kidney, and to play an important pathological role in podocyte diseases [42]. We then sought to identify miRNAs that play a key role in PDE‐induced podocyte developmental toxicity, with a view to developing promising miRNA therapeutics. As predicted and corroborated by the literature [43], miR‐135a‐5p is an identified upstream regulator of KLF4. In this study, dexamethasone significantly elevated miR‐135a‐5p expression in both kidney tissue and MMSCs. Meanwhile, the inhibitory effects of dexamethasone on KLF4 and Nephrin were reversed after miR‐135a‐5p inhibitor treatment was administered to cells and animals. This demonstrated the involvement of high miR‐135a‐5p expression in the inhibition of podocyte differentiation and development by PDE.

Given the pleiotropy of miR‐135a‐5p, KLF4 is unlikely to be its only kidney‐relevant target. Prior work links miR‐135a‐5p to podocyte injury and diabetic renal fibrosis via TRPC1, and miR‐135a‐5p has been shown to promote fibrotic programs in renal cells by targeting SIRT1, implicating additional Ca^2^^+^‐handling and pro‐fibrotic branches that could coexist with the miR‐135a‐5p/KLF4 axis [42, 44, 45]. Moreover, miR‐135a‐5p has been reported to repress PTEN in a 3′UTR‐dependent manner in other contexts, and both PTEN and Wnt/β‐catenin (CTNNB1) signaling are pivotal for podocyte integrity [46]. Future work using unbiased, cell‐type resolved approaches (e.g., AGO2‐RIP/CLIP combined with transcriptomics/spatial profiling) will be valuable to map the broader miR‐135a‐5p regulatory network and its interactions beyond the miR‐135a‐5p/KLF4 axis in PDE.

Notably, miR‐135a‐5p was increased in PBMCs from PDE offspring, suggesting that PDE‐associated miR‐135a‐5p dysregulation may be detectable in accessible peripheral compartments. Although serum/urine miR‐135a‐5p could not be reliably quantified with the current technical setup, and pediatric sampling will require prospective recruitment with adequate sample size and follow‐up, such clinical validation is crucial for confirming miR‐135a‐5p as a fetal‐origin target/biomarker and for building the evidence chain needed to translate mechanistic findings into clinically actionable risk stratification and intervention.

Sex is an important biological variable in fetal programming, and increasing evidence indicates that prenatal glucocorticoid exposure can produce sex‐dependent long‐term outcomes across multiple organ systems [47, 48, 49, 50, 51]. In this study, we extended the analysis to female offspring and found that miR‐135a‐5p and KLF4 were not significantly altered in fetal or adult female kidneys (Figure S2C,D), despite a reduction in podocyte marker expression [20], pointing to sexually dimorphic molecular wiring of PDE responses. This divergence is consistent with reports that glucocorticoid signaling can be sexually dimorphic and may involve crosstalk between GR and sex‐steroid receptors [52, 53]. Notably, estrogen has been shown to antagonize glucocorticoid induction of GILZ [54], raising the possibility that estrogenic signaling partially buffers GR‐dependent programming in females and limits activation of the miR‐135a‐5p/KLF4 axis. Collectively, these findings support the multi‐organ, sex‐dependent programming effects of PDE. The mechanism of sex differences in renal developmental toxicity in offspring rats caused by PDE will be further studied in subsequent work.

Intrauterine programming refers to alterations in fetal gene expression or function caused by an adverse intrauterine environment, the effects of which could persist beyond birth and even throughout life. Studies have shown that aberrant epigenetic modifications are the main mechanism mediating the altered intrauterine programming [55]. Our findings indicate that PDE induces high renal miR‐135a‐5p expression in offspring that persists from intrauterine to adulthood, suggesting that there may be altered intrauterine programming of high miR‐135a‐5p expression. Further exploration also revealed that miR‐135a‐5p promoter region H3K9ac levels in the PDE group were consistently elevated from the intrauterine to the postnatal period. In vitro experiments showed that dexamethasone directly altered the levels of H3K9ac, H3K14ac, and H3K27ac in the miR‐135a‐5p promoter region. These results suggested that hyperacetylation of H3K9 histone in the miR‐135a‐5p promoter region may be the intrauterine programming mechanism that leads to PDE‐induced podocyte developmental toxicity in offspring.

To further explore the molecular mechanism of miR‐135a‐5p intrauterine programming, we predicted that GR could bind to the miR‐135a‐5p promoter region using the JASPAR website, and therefore hypothesized that dexamethasone might act on the miR‐135a‐5p promoter region GRE and recruit various cofactors to synergistically regulate its expression [34]. To confirm the binding of GR to the miR‐135a‐5p promoter region, we utilized a dual luciferase reporter gene assay to confirm that a GRE does exist in the miR‐135a‐5p promoter region and that its expression is directly regulated by dexamethasone.

Histone acetylases or deacetylases affect gene transcription at the epigenetic level through post‐translational modification of histones [56]. P300 is a transcriptional coactivator that can be recruited and bound to the promoter region of target genes by a variety of transcription factors, including GR, and affects gene transcription through histone acetylation modifications [35]. It has been reported that histone acetylase P300 can be involved in the regulation of miR‐135a‐5p expression [57]. In exploring the molecular mechanism of PDE‐induced altered intrauterine programming of miR‐135a‐5p in offspring, we found that dexamethasone not only promoted the intranuclear expression of GR and P300, but also the binding of GR and P300 to the miR‐135a‐5p promoter region. Our results suggested that on the one hand, dexamethasone activates GR into the nucleus of renal MMSCs to directly promote miR‐135a‐5p transcription, and on the other hand, it promotes GR to recruit more P300 to bind to the miR‐135a‐5p promoter region to promote miR‐135a‐5p transcription by regulating histone acetylation.

Although our data support histone acetylation as the major persistent regulatory layer at the miR‐135a‐5p promoter, we cannot exclude contributions from other epigenetic mechanisms—particularly DNA methylation/hydroxymethylation—which should be addressed by systematic epigenomic profiling in future work. Besides, though our data pinpoint GR and P300 at the miR‐135a‐5p promoter, the upstream cues that bias this complex toward enhanced occupancy in PDE remain to be clarified. One plausible layer is stress‐kinase control of GR, as p38 MAPK/JNK signaling can modulate GR function through site‐specific phosphorylation (including links between p38 activation and GR Ser211 phosphorylation, and JNK‐associated GR Ser226 phosphorylation with reduced transcriptional output and enhanced nuclear export) [58, 59]. In parallel, PI3K–Akt signaling can increase P300 coactivator activity; Akt phosphorylation of P300 at Ser1834 has been shown to boost its HAT function and promoter recruitment, providing a straightforward route to the heightened histone acetylation we observe [60]. Finally, GR coactivator assembly is often scaffolded by p160/SRC‐family coactivators, which can recruit secondary coactivators such as CBP/P300, offering an additional mechanism by which PDE could favor GR–P300 loading at selected loci like the miR‐135a‐5p promoter [61]. These possibilities can be tested by profiling GR/P300 phosphorylation states and key upstream kinase pathways in future studies.

Early prevention and treatment of fetal‐derived disease present significant challenges due to unclear etiology and pathogenic mechanisms, and lack of targets for intervention. In recent years, targeting non‐coding RNAs (ncRNAs), including miRNAs and long non‐coding RNAs, has emerged as a promising strategy for the treatment of various diseases [62, 63]. A notable example is the ongoing phase II clinical trial testing anti‐miR‐21 for the treatment of Alport syndrome, a genetic disorder associated with renal dysfunction [64]. This suggests that miRNAs have great potential as therapeutic targets for the prevention and treatment of kidney disease. However, only a handful of miRNA therapies have entered clinical trials, none of which have reached phase III or received FDA approval to date, and several of which have been terminated due to toxicity. In light of this situation, the biotechnology and pharmaceutical industries have focused on the development of two classes of miRNA drugs, miRNA mimics and inhibitors (antagomir or anti‐mir) [65]. miRNA antagomir is a specially chemically modified miRNA antagonist that inhibits miRNA action by binding strongly and competitively to mature miRNAs in vivo, preventing complementary pairing of miRNAs with their target gene mRNAs. In this study, we administered miR‐135a‐5p antagomir to mice at a young age to inhibit the binding of miR‐135a‐5p to target genes and found that it reversed the PDE‐induced low expression of KLF4, Nephrin, and Podocin at PW7 and PW28, and partially reversed glomerulosclerosis (PW28) in PDE offspring mice. The lack of a significant improvement in serum creatinine is not unexpected because creatinine is a relatively insensitive readout of global filtration, is affected by multiple extra‐renal determinants, and therefore does not necessarily mirror partial or compartment‐restricted structural repair [66]. Even without a complete functional rescue, our data are intriguing in that they suggest early‐life miRNA modulation can exert durable effects on the later trajectory of fetal‐origin disease. Indeed, prior work indicates that adverse intrauterine exposures typically operate through multiple pathways, organs, and targets [67]. Accordingly, PDE may imprint broader and more persistent programming effects (e.g., reduced nephron endowment and long‐term hemodynamic stress) [68] that may not be fully reversible by targeting a single podocyte‐centered axis. Moreover, loss of renal function also correlates with “extra‐glomerular” lesions—including tubular atrophy, interstitial fibrosis, and microvascular alterations [69]—raising the possibility that residual tubular/interstitial or endothelial injury contributes to the incomplete recovery of renal function. Prevention and treatment of fetal‐origin kidney disease therefore, remains an important challenge. Nevertheless, our findings support miR‐135a‐5p as a potentially tractable target for mitigating podocyte developmental impairment–driven glomerulosclerosis.

While early‐life miR‐135a‐5p antagomir treatment restored podocyte markers and mitigated glomerulosclerosis, the functional evaluation was limited to creatinine‐based measures. Because creatinine may be insensitive to partial or compartment‐restricted improvement, future studies should incorporate proteinuria, additional filtration markers (BUN/cystatin C), and ideally direct GFR measurement to better define therapeutic benefit. Beyond efficacy, the long‐term safety and specificity of early‐life anti‐miR strategies warrant careful consideration. Antisense miRNA oligonucleotides can carry off‐target risk through unintended interactions with non‐target endogenous nucleic acids and through immunomodulatory effects, including reported interference with innate immune TLR sensing in a context‐ and chemistry‐dependent manner [70, 71]. In the present study, ALT/AST and TG levels were not altered in adulthood after early‐life miR‐135a‐5p inhibition (Figure S2E–G), providing an initial systemic safety signal; however, broader multi‐organ assessment, biodistribution studies, cytokine profiling, and unbiased off‐target transcriptomic analyses will be required to more comprehensively establish long‐term safety.

A limitation of this study is that, while we focused on podocytes as a developmentally vulnerable and poorly regenerative trigger of glomerulosclerosis, PDE may induce broader multi‐compartment renal programming. Consistent with this possibility, our preliminary screening detected changes beyond podocyte markers (e.g., Fn1 downregulation and Edn1 upregulation), whereas tubular markers (Lrp2) and an injury‐associated transcript (Lcn2) showed no overt alterations at the examined time points (Figure S5H). These bulk measurements do not exclude subtle, region‐specific tubular dysfunction or microvascular/tubulointerstitial remodeling that could contribute to long‐term functional impairment. Future cell‐type resolved analyses (e.g., single‐cell/single‐nucleus RNA‐seq and spatial transcriptomics) will be important to define the contributions and interactions of distinct renal cell populations in PDE‐induced developmental nephrotoxicity.

In conclusion, we demonstrate that prenatal dexamethasone exposure (PDE) programs podocyte developmental toxicity and predisposes offspring to glomerulosclerosis. Mechanistically, dexamethasone enhances binding of GR to the miR‐135a‐5p promoter region and promotes P300 recruitment, increasing histone acetylation and thereby establishing sustained miR‐135a‐5p upregulation. Elevated miR‐135a‐5p suppresses KLF4, impairing podocyte development with consequences that persist from fetal life into adulthood. Notably, early‐life miR‐135a‐5p antagonism partially rescues podocyte injury and mitigates glomerulosclerosis, highlighting miR‐135a‐5p as a tractable target for preventing fetal‐origin glomerular disease induced by adverse prenatal environments.

## 4 Materials and Methods

### 4.1 Animals and Treatment

SPF‐grade Wistar rats and C57BL/6 mice were purchased from the Hubei Provincial Center for Disease Control and Prevention. Female rats weighed 200 ± 20 g, and male rats weighed 280 ± 20 g. Female mice weighed 21 ± 3 g, and male mice weighed 24 ± 3 g. Animal experiments were performed at the Center for Animal Experiments/Animal Biosafety Level–III Laboratory of Wuhan University (room temperature: 18–22°C; humidity: 40%–60%; light time: 12 h of darkness with alternating lights). The laboratory center has been accredited by the Association for Assessment and Accreditation of Laboratory Animal Care International (AAALAC International). After one week of acclimatization, the animals were mated daily at 18:00 in a 2:1 ratio of females to males, and the vaginal smears were observed under a microscope at 6:00 on the following day. If sperm were visible in the microscopic examination, it was recorded as GD0. Conceived rats were randomly divided into the PDE group and the control group.

Clinically, antenatal dexamethasone is used not only in the standard short‐course regimen for fetal lung maturation, but also in the management of pregnancies with persistent preterm risk due to obstetric complications such as multiple gestation or placenta previa–related bleeding, where uncertainty in the timing of delivery can lead to cumulative/prolonged exposure. Accordingly, our PDE model was designed to approximate this chronic exposure scenario in mid‐to‐late gestation. We administered dexamethasone to pregnant Wistar rats on GD9 to establish an animal model of PDE. Based on a dose‐conversion relationship between humans and rats (body surface area ratio of human: rat = 1/6.17) [72], rats were subcutaneously injected with dexamethasone at a dose of 0.2 mg/kg·d, which is equivalent to 0.03 mg/kg·d administered in humans, and this is slightly lower than the clinical therapeutic dose for pregnant women (0.05–0.2 mg/kg·d) [73, 74]. Dexamethasone (0.2 mg/kg⋅d) was injected subcutaneously in the PDE group, and an equal volume of saline was given to the control group starting at GD9. We selected only pregnant rats with litter sizes between 10 and 14 as the experimental group.

Fetal rat experiment: Each group was randomly anesthetized with isoflurane to execute GD20 partially pregnant rats (*n* = 12) in the PDE group and pregnant rats (*n* = 12) in the control group, and then the fetal kidneys were taken and fixed with 4% formaldehyde fixative or electron microscope fixative, or placed in the stored at −80°C for preservation and examination.

Postnatal offspring rat experiment: Pregnant rats were delivered naturally. After weaning at PW4, two males were randomly selected from each litter of offspring rats in the PDE group (*n* = 12) and the control group (*n* = 12), and were fed until PW6 and PW28, respectively. Isoflurane anesthesia was used to execute the rats, and kidney tissues were obtained.

Mice offer practical advantages for early‐life repeated dosing and long‐term follow‐up (breeding cycle, cohort size, and cost), and miRNA‐intervention reagents (chemistry, dosing references, and safety data) are more established in C57BL/6. Therefore, the mouse study provides cross‐species support and proof‐of‐concept for early intervention. miR‐135a‐5p intervention experiment: Pregnant C57BL/6 mice were randomized and divided into PDE and control groups. Mice in the PDE group were injected subcutaneously with dexamethasone 0.4 mg/kg, and the control group was injected with an equal amount of saline. After delivery of pregnant mice in the PDE group, one male offspring from each litter was selected to receive intraperitoneal injections of miR‐135a‐5p antagomir every 3 days, during PW1 through PW5. One offspring was taken from each litter of the control group, the PDE group, and the PDE+miR‐135a‐5p antagomir group (*n* = 8), and was anesthetized and executed at PW6 and PW28, respectively, thereby obtaining specimens.

### 4.2 Chemicals and Reagents

Dexamethasone sodium phosphate injection (No. H42021492) was purchased from Huangzhong Pharmaceutical Co., Ltd. (China). RIPA Lysis Buffer was purchased from Beyotime Biotechnology (China). Primary antibodies such as rabbit IgG (No. ab124505) and rabbit anti‐P300 (No. ab14984) were made by Abcam (UK), and rabbit anti‐KLF4 (NBP2‐24749) was obtained from Novus (US). Mouse anti‐GR (No.sc‐12763) and mouse anti‐Nephrin (No. sc‐376522) were purchased from Santa Cruz (US). Rabbit anti‐H3K9 (No. A7255), rabbit anti‐H3K14 (No. A7254), rabbit anti‐H3K27 (No. A7253), and mouse anti‐GAPDH (No. AC001) were obtained from ABclonal Biotechnology Inc. (China). Proteinase K (No. ST533) and TSA (No. S58880) were purchased from Kerui Biotechnology (China). Wnt4 (475‐WNT‐005) was obtained from B&D Systems (US). P300 siRNA, GR siRNA, and KLF4 overexpression plasmid were purchased from GenePharma Co., Ltd. (China). Dual–Luciferase Reporter Assay System (E1910) was provided by Promega (US). Isoflurane was obtained from Baxter Healthcare Co. (Deerfield, IL, USA). Trizol Reagent (No. 15596026) was provided by Thermo Fisher Scientific Co., Ltd. (USA). RT‐qPCR kits were purchased from Takara Biotech Co., Ltd. (China). Oligonucleotide primers were synthesized by Shanghai Shenggong Biotech Co., Ltd. (China). Lipofectamine 3000 Reagent and P3000 Reagent were provided by Invitrogen (USA).

All other chemicals and reagents were of analytical grade.

### 4.3 Total RNA Extract and RT‐qPCR

Total RNA from the homogenized tissue or cells was extracted using Trizol. After the addition of chloroform and centrifugation, the sample was divided into an aqueous and an organic layer. After collecting the upper aqueous layer, the RNA was separated out using isopropanol. Then the RNA precipitates were washed using 75% ethanol and dissolved in RNAase‐free water. Using the protocol from the Applied Biosystems TaqMan Reverse Transcription Kit, single‐stranded cDNA was created from a 1 µg sample of total RNA. The relative mRNA expression levels of the target genes were detected by the RT‐qPCR kit and normalized to GAPDH using the 2^‐ΔΔCT^ method. The sequences of the primers used in the experiment are detailed in Table S1.

### 4.4 Western Blot

Kidney tissue homogenates or cells were lysed for protein in RIPA buffer and then centrifuged at 12,000 g for 10 min at 4°C to collect the supernatant. The lysate was mixed with fivefold up‐sampling buffer and boiled at 100°C for 10 min. Samples containing equal amounts of protein were separated by 10% SDS‐PAGE and transferred to a polyvinylidene difluoride membrane. To prevent nonspecific binding, the membranes were incubated in TBST containing 5% nonfat dry milk for 120 min at room temperature. The membranes were then incubated with primary antibodies against Nephrin (1:500), KLF4 (1:1000), GR (1:2000), P300 (1:1000), GAPDH (1:500), or H3 (1:500) at 4°C overnight. After incubation with enzyme‐labeled secondary antibody (1:5000) for 1 h, the signals were detected with ECL reagent. For comparison of protein density levels between groups, samples were normalized by GPADH or H3.

### 4.5 Histological and Immunofluorescence

Kidney tissue sections were stained with hematoxylin‐eosin (HE) or periodic acid‐schiff (PAS) reagent to assess the GSI, as previously described [75, 76]. Glomerulosclerosis was semi‐quantitatively graded in a blinded manner using a five‐grade scoring system: 0, no sclerosis; 1, sclerosis involving <25% of the glomerular tuft area; 2, 25%–50%; 3, 50%–75%; 4, >75% (or global sclerosis when applicable). A minimum of glomeruli per animal was scored at ×400 magnification by two independent observers, and the mean score was used for analysis. The GSI was calculated as: GSI = [(1×N1)+(2×N2)+(3×N3)+(4×N4)] / (N0+N1+N2+N3+N4).

For immunofluorescence, paraffin‐embedded kidney tissue sections were deparaffinized and rehydrated in xylene. After boiling in Tris‐EDTA (pH 9) for 10 min, the sections were rinsed 3 times in PBS, incubated in 3% H_2_O_2_ for 10 min, and blocked in 5% BSA for 20 min. The sections were then incubated with the primary antibody for approximately 24 h at 4°C. The sections were then washed 3 times in PBS and shaken for 5 min each time on a decolorizing shaker. The corresponding fluorescent secondary antibody (1:400) was added and incubated for 1 h at room temperature away from light. Sections were incubated in nuclear stain (DAPI, 1:500) for 10 min. Images were captured using an Olympus AH‐2 light microscope and analyzed using Olympus software.

### 4.6 Cell Culture and Treatment

Kidneys from GD14 fetuses were sheared and digested with type 1 collagenase, and then filtered through a 70 µm filter, resulting in the isolation of MMSCs for culture [21]. Briefly, fetal kidneys were dissected under sterile conditions, rinsed in calcium/magnesium‐free PBS, and minced into small fragments. Tissues were enzymatically dissociated using collagenase (0.2%, 37°C, 30 min), followed by trypsin/EDTA (0.25% trypsin, 0.02% EDTA, 37°C, 10–15 min) with gentle agitation. The cell suspension was filtered through a 70 µm strainer, centrifuged, and resuspended in DMEM/F12 supplemented with 10% fetal bovine serum and penicillin/streptomycin. Cells were maintained at 37°C in 5% CO_2_. After 24 h, non‐adherent cells were removed, and adherent cells were further expanded; passages 2–3 were used for downstream assays. To verify cell identity, MMSCs were characterized by flow cytometry for lineage and mesenchymal markers. Cells were positive for metanephric/renal progenitor‐associated markers (e.g., PAX2 and/or other renal developmental factors) and mesenchymal markers (e.g., VIM, FN1, and CD44), while being negative for hematopoietic/endothelial markers (e.g., CD34). In addition, the renal progenitor‐like molecular profile reported for embryonic kidney‐derived clonogenic populations (including expression of kidney developmental transcription factors such as WT1, SIX2, SALL1, and related genes) was used to support the metanephric origin and lineage relevance of the isolated cells.

The cells were seeded homogeneously in cell culture plates for 24 h before drug treatment. Then cells were treated with different concentrations of dexamethasone, and collected for subsequent analysis. The in vitro dose was based on previous measurements in our laboratory, which showed a fetal blood dexamethasone concentration of 267 nM in a 0.2 mg/kg/ day PDE rat model [11]. Therefore, we used 20, 100, and 500 nM dexamethasone in vitro to cover this exposure range. In the experiment to verify the mechanism of action of dexamethasone, cells were treated with a combination of miR‐135a‐5p inhibitor, KLF4 overexpression plasmids, GR siRNA, or P300 siRNA for 24 h, and then continued to be treated with dexamethasone before the subsequent analysis (the siRNA sequences used in the experiment are detailed in Table S2). For the podocyte differentiation model, we administered Wnt4 (100 ng/mL) on MMSCs for 72 h to promote their differentiation to podocytes.

### 4.7 Ultrastructural Observation

Freshly extracted fetal kidneys and adult kidney tissues (operated on ice) were cut into small pieces of approximately 1 mm^3^ in size, pre‐fixed in 2.5% glutaraldehyde solution (0.2 M dimethyl arsenate buffer, pH 7.4) for a few hours, and then fixed with osmium acid for approximately 1.5 h. The tissues were rinsed with distilled water and then sequentially added to ethanol (50%, 70%, and 90%), ethanol‐acetone (1:1), and acetone (90%, 100%) for dehydration, each period taking 15–20 min. The specimens were embedded with epoxy resin, after which they were cut into 50 nm slices using an ultrathin sectioning machine. Finally, the specimens were double‐stained with 1.25% uranyl acetate and 0.4% lead citrate. Transmission electron microscopy was used to observe the morphology such as the number and structure of foot processes.

### 4.8 Chromatin Immunoprecipitation (ChIP) Assay

Kidneys and MMSCs were fixed in 1% formaldehyde on ice for 10 min. The reaction was stopped for 5 min by adding glycine at a final concentration of 125 mM on ice. After centrifugation, the sediments were resuspended in SDS lysis buffer containing protease inhibitors and incubated on ice for 10 min. Next, the lysate was centrifuged at 12,000 rpm for 10 min at 4°C, and the supernatant was transferred to a new tube and diluted with ChIP dilution buffer. Chromatin was incubated with anti‐H3K9, anti‐H3K14, or anti‐H3K27 antibodies for at least 6 h on a rotator at 4°C; bovine serum albumin (BSA)‐treated protein G microbeads were added to reduce nonspecific binding. Immunoprecipitated DNA‐protein complexes linked to protein G beads were collected by centrifugation and washed sequentially with low salt, high salt, LiCl immunocomplexes, and Tris‐EDTA wash buffer. The DNA‐protein complexes were eluted with freshly prepared elution buffer (1% SDS, 0.1 M NaHCO3). Each elution was repeated twice. The samples were then placed in a water bath at 65°C for 24 h to reverse formaldehyde cross‐linking and the proteins were digested with 200 µg/mL proteinase K. The DNA was subsequently purified using the QIA quick PCR purification kit according to the manufacturer's protocol.

### 4.9 Luciferase Reporter Assay

The miR‐135a‐5p promoter fragment was inserted into the firefly luciferase expression sequence to construct a reporter gene plasmid. The GR overexpression plasmid was co‐transfected with the firefly luciferase reporter gene plasmid or the Renilla luciferase internal reference gene plasmid, respectively (6–8 wells per set of replicates). The cells were harvested and lysed 24 h later. After the addition of the respective luciferase substrates, the samples were put into a luminometer to read the luminescence values.

### 4.10 Co‐Immunoprecipitation

Cells were lysed on ice and broken by sonication. Protein A and Protein G were added to the cell lysate and incubated at 4°C with rotation. An amount of P300 antibody or IgG antibody was added to the supernatant and mixed overnight. Then an amount of protein A and protein G was added to the supernatant, gently mixed at 4°C and incubated for 3 h. The interacting protein complexes were separated from the beads using elution buffer. Western blotting assay was then performed.

### 4.11 Statistical Analysis

Data were analyzed using IBM SPSS Statistics 20 (SPSS Science Inc, Chicago, IL, USA) and GraphPad Prism 8.0 (GraphPad Software, La Jolla, CA, USA). All datasets were tested for normality using the Shapiro–Wilk test and for homogeneity of variance using Levene's test prior to inferential statistics. Two‐group comparisons were performed using two‐tailed unpaired Student's *t*‐tests. For comparisons among multiple groups, one‐way ANOVA was applied, followed by post hoc testing selected according to the study design: Dunnett's test for comparisons versus the control group and Tukey's test for pairwise comparisons across all groups. Repeated‐measures data were analyzed by repeated‐measures ANOVA, with Bonferroni correction applied when appropriate. When assumptions of normality and/or equal variance were violated, nonparametric alternatives were used (e.g., Mann–Whitney U test or Kruskal–Wallis test with appropriate multiple‐comparison adjustment). Data are presented as mean ± SEM, and *p* < 0.05 was considered statistically significant.

### 4.12 Ethics Declaration

The experiment was performed in strict accordance with the principles and guidelines of the China Animal Welfare Committee for the use and care of animal experiments, and the operating procedures were approved by the Animal Experiment Ethics Committee of Wuhan University Medical College (license No. WP20210061).

## Author Contributions

The study was designed and conceived by Y.A. The experiments were conducted by H.C., X.Z., Y.Z., Z.X., H.H., Y.L., and T.Y. The manuscript was written by X.Z. and the experimental data were prepared and analyzed by X.Z. and H.C. H.W., Y.A., and Y.Z. gave important input in the design and guidance of the experiment.

## Funding

National Key Research and Development Program of China (No. 2020YFA0803900), National Natural Science Foundation of China (No. 81872943), Hubei Provincial Key Laboratory Open Project‐Seed Fund Program (No. 2025KFZ025), and Natural Science Foundation of Hubei Province (No. 2023AFB238)

## Conflicts of Interest

The authors declare no conflicts of interest.

## Supporting information




**Supporting File**: advs74128‐sup‐0001‐SuppMat.docx.

## Data Availability

The data that support the findings of this study are available from the corresponding author upon reasonable request.

## References

[1] J. C. Durán‐Álvarez , B. Prado , R. Zanella , M. Rodríguez , and S. Díaz , “Wastewater Surveillance of Pharmaceuticals during the COVID‐19 Pandemic in Mexico City and the Mezquital Valley: a Comprehensive Environmental Risk Assessment,” Science of The Total Environment 900 (2023): 165886, 10.1016/j.scitotenv.2023.165886. 37524191

[2] S. Liu , G. G. Ying , L. J. Zhou , R. Q. Zhang , Z. F. Chen , and H. J. Lai , “Steroids in a Typical Swine Farm and Their Release into the Environment,” Water Research 46, no. 12 (2012): 3754–3768, 10.1016/j.watres.2012.04.006. 22591816

[3] N. Rakonjac , S. van der Zee , L. Wipfler , et al., “An Analytical Framework on the Leaching Potential of Veterinary Pharmaceuticals: a Case Study for the Netherlands,” Science of The Total Environment 859, no. Pt 2 (2023): 160310, 10.1016/j.scitotenv.2022.160310. 36410490

[4] C. A. Crowther , C. J. McKinlay , P. Middleton , and J. E. Harding , “Repeat Doses of Prenatal Corticosteroids for Women at Risk of Preterm Birth for Improving Neonatal Health Outcomes,” Cochrane Database of Systematic Reviews , no. 7 (2015): CD003935, 10.1002/14651858.CD003935.pub4. 26142898 PMC7104525

[5] J. P. Vogel , J. P. Souza , A. M. Gülmezoglu , et al., “Use of Antenatal Corticosteroids and Tocolytic Drugs in Preterm Births in 29 Countries: an Analysis of the WHO Multicountry Survey on Maternal and Newborn Health,” The Lancet 384, no. 9957 (2014): 1869–1877, 10.1016/s0140-6736(14)60580-8. 25128271

[6] R. D'Souza , R. Ashraf , H. Rowe , et al., “Pregnancy and COVID ‐19: Pharmacologic Considerations,” Ultrasound in Obstetrics & Gynecology 57, no. 2 (2021): 195–203, 10.1002/uog.23116. 32959455 PMC7537532

[7] K. Ninan , A. Gojic , Y. Wang , et al., “The Proportions of Term or Late Preterm Births after Exposure to Early Antenatal Corticosteroids, and Outcomes: Systematic Review and Meta‐Analysis of 1.6 Million Infants,” Bmj 382 (2023): 076035, 10.1136/bmj-2023-076035. PMC10394681 37532269

[8] V. G. Moisiadis and S. G. Matthews , “Glucocorticoids and Fetal Programming Part 1: Outcomes,” Nature Reviews Endocrinology 10, no. 7 (2014): 391–402, 10.1038/nrendo.2014.73. 24863382

[9] ACOG Committee Opinion ,“Committee Opinion No. 475: Antenatal Corticosteroid Therapy for Fetal Maturation,” Obstetrics & Gynecology 117, no. 2 Pt 1 (2011): 422–424, 10.1097/AOG.0b013e31820eee00. 21252775

[10] M. Liu and Y. Zhang , “Discursive Constructions of Scientific (Un)Certainty about the Health Risks of China's Air Pollution: a Corpus‐Assisted Discourse Study,” Language & Communication 60 (2018): 1–10, 10.1016/j.langcom.2018.01.006.

[11] F. Lv , Y. Wan , Y. Chen , et al., “Prenatal Dexamethasone Exposure Induced Ovarian Developmental Toxicity and Transgenerational Effect in Rat Offspring,” Endocrinology 159, no. 3 (2018): 1401–1415, 10.1210/en.2018-00044. 29370380

[12] X. Zhang , Y. Shang‐Guan , J. Ma , et al., “Mitogen‐Inducible Gene‐6 Partly Mediates the Inhibitory Effects of Prenatal Dexamethasone Exposure on Endochondral Ossification in Long Bones of Fetal Rats,” British Journal of Pharmacology 173, no. 14 (2016): 2250–2262, 10.1111/bph.13506. 27128203 PMC4919583

[13] S. J. Shankland , “The Podocyte's Response to Injury: Role in Proteinuria and Glomerulosclerosis,” Kidney International 69, no. 12 (2006): 2131–2147, 10.1038/sj.ki.5000410. 16688120

[14] T. Sakairi , Y. Abe , H. Kajiyama , et al., “Conditionally Immortalized human Podocyte Cell Lines Established from Urine,” American Journal of Physiology‐Renal Physiology 298, no. 3 (2010): F557–F567, 10.1152/ajprenal.00509.2009. 19955187 PMC2838606

[15] V. A. Luyckx and B. M. Brenner , “Low Birth Weight, Nephron Number, and Kidney Disease,” Kidney International 68, no. 97 (2005): S68–S77, 10.1111/j.1523-1755.2005.09712.x. 16014104

[16] J. B. Hodgin , M. Rasoulpour , G. S. Markowitz , and V. D. D'Agati , “Very Low Birth Weight Is a Risk Factor for Secondary Focal Segmental Glomerulosclerosis,” Clinical Journal of the American Society of Nephrology 4, no. 1 (2009): 71–76, 10.2215/CJN.01700408. 19019999 PMC2615706

[17] G. Conti , D. De Vivo , C. Fede , et al., “Low Birth Weight Is a Conditioning Factor for Podocyte Alteration and Steroid Dependance in Children with Nephrotic Syndrome,” Journal of Nephrology 31, no. 3 (2018): 411–415, 10.1007/s40620-018-0473-7. 29350347

[18] J. Dötsch , M. Alejandre‐Alcazar , R. Janoschek , E. Nüsken , L. T. Weber , and K. D. Nüsken , “Perinatal Programming of Renal Function,” Current Opinion in Pediatrics 28, no. 2 (2016): 188–194, 10.1097/mop.0000000000000312. 26963856

[19] L. F. Almeida , S. S. Tofteng , K. Madsen , and B. L. Jensen , “Role of the Renin–angiotensin System in Kidney Development and Programming of Adult Blood Pressure,” Clinical Science 134, no. 6 (2020): 641–656, 10.1042/cs20190765. 32219345

[20] X. Zhao , Z. Wang , Z. Xia , et al., “Dexamethasone Induces Transgenerational Inheritance of Fetal‐Derived Glomerulosclerosis Phenotype in Offspring through GR/DNMT3a Mediated Alterations of the lncRNA‐Meg3/Notch Signaling Pathway,” Cell Communication and Signaling 23, no. 1 (2025): 345, 10.1186/s12964-025-02346-1. 40676611 PMC12273400

[21] B. Li , Y. Zhu , H. Chen , et al., “Decreased H3K9ac Level of AT2R Mediates the Developmental Origin of Glomerulosclerosis Induced by Prenatal Dexamethasone Exposure in Male Offspring Rats,” Toxicology 411 (2019): 32–42, 10.1016/j.tox.2018.10.013. 30359671

[22] A. Greka and P. Mundel , “Cell Biology and Pathology of Podocytes,” Annual Review of Physiology 74 (2012): 299–323, 10.1146/annurev-physiol-020911-153238. PMC3600372 22054238

[23] M. Nagata , “Podocyte Injury and Its Consequences,” Kidney International 89, no. 6 (2016): 1221–1230, 10.1016/j.kint.2016.01.012. 27165817

[24] A. Z. Rosenberg and J. B. Kopp , “Focal Segmental Glomerulosclerosis,” Clinical Journal of the American Society of Nephrology 12, no. 3 (2017): 502–517, 10.2215/cjn.05960616. 28242845 PMC5338705

[25] E. Huntzinger and E. Izaurralde , “Gene Silencing by microRNAs: Contributions of Translational Repression and mRNA Decay,” Nature Reviews Genetics 12, no. 2 (2011): 99–110, 10.1038/nrg2936. 21245828

[26] Y. L. Phua , J. Y. S. Chu , A. K. Marrone , A. J. Bodnar , S. Sims‐Lucas , and J. Ho , “Renal Stromal miRNAs Are Required for Normal Nephrogenesis and Glomerular Mesangial Survival,” Physiological Reports 3, no. 10 (2015): 12537, 10.14814/phy2.12537. PMC4632944 26438731

[27] N. Mahtal , O. Lenoir , C. Tinel , D. Anglicheau , and P. L. Tharaux , “MicroRNAs in Kidney Injury and Disease,” Nature Reviews Nephrology 18, no. 10 (2022): 643–662, 10.1038/s41581-022-00608-6. 35974169

[28] K. Hayashi , H. Sasamura , M. Nakamura , et al., “KLF4‐Dependent Epigenetic Remodeling Modulates Podocyte Phenotypes and Attenuates Proteinuria,” Journal of Clinical Investigation 124, no. 6 (2014): 2523, 10.1172/jci69557. 24812666 PMC4089466

[29] P. Sidaway , “KLF4 promotes Podocyte Differentiation,” Nature Reviews Nephrology 10, no. 7 (2014): 362, 10.1038/nrneph.2014.96. 24861086

[30] P. Periyasamy , K. Liao , Y. H. Kook , et al., “Cocaine‐Mediated Downregulation of miR‐124 Activates Microglia by Targeting KLF4 and TLR4 Signaling,” Molecular Neurobiology 55, no. 4 (2018): 3196–3210, 10.1007/s12035-017-0584-5. 28478506 PMC5673594

[31] G. Vares , S. Sai , B. Wang , A. Fujimori , M. Nenoi , and T. Nakajima , “Progesterone Generates Cancer Stem Cells through Membrane Progesterone Receptor‐Triggered Signaling in Basal‐Like human Mammary Cells,” Cancer Letters 362, no. 2 (2015): 167–173, 10.1016/j.canlet.2015.03.030. 25819032

[32] E. Y. van Battum , M. G. Verhagen , V. R. Vangoor , et al., “An Image‐Based miRNA Screen Identifies miRNA‐135s as Regulators of CNS Axon Growth and Regeneration by Targeting Krüppel‐Like Factor 4,” The Journal of Neuroscience 38, no. 3 (2018): 613–630, 10.1523/jneurosci.0662-17.2017. 29196317 PMC6596187

[33] C. Chen , X. Mao , C. Cheng , et al., “miR‐135a Reduces Osteosarcoma Pulmonary Metastasis by Targeting both BMI1 and KLF4,” Frontiers in Oncology 11 (2021): 620295, 10.3389/fonc.2021.620295. 33828977 PMC8019936

[34] D. J. Mangelsdorf , C. Thummel , M. Beato , et al., “The Nuclear Receptor Superfamily: The Second Decade,” Cell 83, no. 6 (1995): 835–839, 10.1016/0092-8674(95)90199-x. 8521507 PMC6159888

[35] W. Wang , C. Guo , W. Li , et al., “Involvement of GR and p300 in the Induction of H6PD by Cortisol in Human Amnion Fibroblasts,” Endocrinology 153, no. 12 (2012): 5993–6002, 10.1210/en.2012-1531. 23125313 PMC3512073

[36] H. Hamatani , T. Sakairi , H. Ikeuchi , et al., “TGF‐β1 Alters DNA Methylation Levels in Promoter and Enhancer Regions of the WT1 Gene in human Podocytes,” Nephrology 24, no. 5 (2019): 575–584, 10.1111/nep.13411. 29851165

[37] S. C. Done , M. Takemoto , L. He , et al., “Nephrin Is Involved in Podocyte Maturation but Not Survival during Glomerular Development,” Kidney International 73, no. 6 (2008): 697–704, 10.1038/sj.ki.5002707. 18046313

[38] G. M. Ghiggeri , M. Gigante , and A. Di Donato , “Constitutional Nephrin Deficiency in Conditionally Immortalized Human Podocytes Induced Epithelial‐Mesenchymal Transition, Supported by β ‐Catenin/NF‐kappa B Activation: a Consequence of Cell Junction Impairment?,” International Journal of Nephrology 2013 (2013): 1–15, 10.1155/2013/457490. PMC3874297 24392227

[39] V. G. Puelles and M. J. Moeller , “Postnatal Podocyte Gain: Is the Jury Still Out?,” Seminars in Cell & Developmental Biology 91 (2019): 147–152, 10.1016/j.semcdb.2018.07.007. 31178004

[40] J. A. Pace , R. Bronstein , Y. Guo , et al., “Podocyte‐Specific KLF4 Is Required to Maintain Parietal Epithelial Cell Quiescence in the Kidney,” Science advances 7, no. 36 (2021): abg6600, 10.1126/sciadv.abg6600. PMC8442927 34516901

[41] Y. Zhu , H. Chen , X. Zhao , et al., “Decreased H3K9ac Level of KLF4 Mediates Podocyte Developmental Toxicity Induced by Prenatal Caffeine Exposure in Male Offspring Rats,” Toxicology Letters 314 (2019): 63–74, 10.1016/j.toxlet.2019.07.011. 31306741

[42] X. Yang , D. Wu , H. Du , F. Nie , X. Pang , and Y. Xu , “MicroRNA‐135a Is Involved in Podocyte Injury in a Transient Receptor Potential Channel 1‐Dependent Manner,” International Journal of Molecular Medicine 40, no. 5 (2017): 1511–1519, 10.3892/ijmm.2017.3152. 28949388 PMC5627871

[43] S. Yao , C. Tian , Y. Ding , et al., “Down‐Regulation of Krüppel‐Like Factor‐4 by microRNA‐135a‐5p Promotes Proliferation and Metastasis in Hepatocellular Carcinoma by Transforming Growth Factor‐β1,” Oncotarget 7, no. 27 (2016): 42566–42578, 10.18632/oncotarget.9934. 27302923 PMC5173156

[44] J. Zhang , L. Zhang , D. Zha , and X. Wu , “Inhibition of miRNA‑135a‑5p Ameliorates TGF‑β1‑Induced human Renal Fibrosis by Targeting SIRT1 in Diabetic Nephropathy,” International Journal of Molecular Medicine 46, no. 3 (2020): 1063–1073, 10.3892/ijmm.2020.4647. 32705273 PMC7387088

[45] F. He , F. Peng , X. Xia , et al., “MiR‐135a Promotes Renal Fibrosis in Diabetic Nephropathy by Regulating TRPC1,” Diabetologia 57, no. 8 (2014): 1726–1736, 10.1007/s00125-014-3282-0. 24908566

[46] J. Wang , L. Zhang , W. Jiang , et al., “MicroRNA‐135a Promotes Proliferation, Migration, Invasion and Induces Chemoresistance of Endometrial Cancer Cells,” European Journal of Obstetrics & Gynecology and Reproductive Biology: X 5 (2020): 100103, 10.1016/j.eurox.2019.100103. 32021975 PMC6994408

[47] Z. Chen , Z. Zhao , Y. Li , et al., “Course‐, Dose‐, and Stage‐Dependent Toxic Effects of Prenatal Dexamethasone Exposure on Fetal Articular Cartilage Development,” Toxicology Letters 286 (2018): 1–9, 10.1016/j.toxlet.2018.01.008. 29329878

[48] Y. Dai , Z. Lu , Y. Peng , et al., “Serpina3c protects against metabolic dysfunction‐associated steatotic liver disease in offspring induced by prenatal prednisone exposure,” Signal Transduction and Targeted Therapy (2026), 10.1038/s41392-025-02569-1. PMC12917113 41708585

[49] K. E. Murphy , A. R. Willan , M. E. Hannah , et al., “Effect of Antenatal Corticosteroids on Fetal Growth and Gestational Age at Birth,” Obstetrics & Gynecology 119, no. 5 (2012): 917–923, 10.1097/AOG.0b013e31825189dc. 22525902

[50] R. J. Wapner , Y. Sorokin , L. Mele , et al., “Long‐Term Outcomes after Repeat Doses of Antenatal Corticosteroids,” New England Journal of Medicine 357, no. 12 (2007): 1190–1198, 10.1056/NEJMoa071453. 17881751

[51] A. K. Elfayomy and S. M. Almasry , “Effects of a Single Course versus Repeated Courses of Antenatal Corticosteroids on Fetal Growth, Placental Morphometry and the Differential Regulation of Vascular Endothelial Growth Factor,” Journal of Obstetrics and Gynaecology Research 40, no. 11 (2014): 2135–2145, 10.1111/jog.12466. 25163747

[52] J. Kroon , A. M. Pereira , and O. C. Meijer , “Glucocorticoid Sexual Dimorphism in Metabolism: Dissecting the Role of Sex Hormones,” Trends in Endocrinology & Metabolism 31, no. 5 (2020): 357–367, 10.1016/j.tem.2020.01.010. 32037025

[53] M. Quinn , S. Ramamoorthy , and J. A. Cidlowski , “Sexually Dimorphic Actions of Glucocorticoids: beyond Chromosomes and Sex Hormones,” Annals of the New York Academy of Sciences 1317 (2014): 1–6, 10.1111/nyas.12425. 24739020 PMC12455521

[54] S. Whirledge and J. A. Cidlowski , “Estradiol Antagonism of Glucocorticoid‐Induced GILZ Expression in Human Uterine Epithelial Cells and Murine Uterus,” Endocrinology 154, no. 1 (2013): 499–510, 10.1210/en.2012-1748. 23183181 PMC3529382

[55] M. F. Fraga and M. Esteller , “Epigenetics and Aging: the Targets and the Marks,” Trends in Genetics 23, no. 8 (2007): 413–418, 10.1016/j.tig.2007.05.008. 17559965

[56] M. J. Hadden and A. Advani , “Histone Deacetylase Inhibitors and Diabetic Kidney Disease,” International Journal of Molecular Sciences 19, no. 9 (2018): 2630, 10.3390/ijms19092630. 30189630 PMC6165182

[57] R. B. Selvi , A. Swaminathan , S. Chatterjee , et al., “Inhibition of p300 Lysine Acetyltransferase Activity by luteolin Reduces Tumor Growth in Head and Neck Squamous Cell Carcinoma (HNSCC) Xenograft Mouse Model,” Oncotarget 6, no. 41 (2015): 43806–43818, 10.18632/oncotarget.6245. 26517526 PMC4791268

[58] L. Zeyen , O. M. Seternes , and I. Mikkola , “Crosstalk between p38 MAPK and GR Signaling,” International Journal of Molecular Sciences 23, no. 6 (2022): 3322, 10.3390/ijms23063322. 35328742 PMC8953609

[59] A. L. Miller , M. S. Webb , A. J. Copik , et al., “p38 Mitogen‐Activated Protein Kinase (MAPK) Is a Key Mediator in Glucocorticoid‐Induced Apoptosis of Lymphoid Cells: Correlation between p38 MAPK Activation and Site‐Specific Phosphorylation of the Human Glucocorticoid Receptor at Serine 211,” Molecular Endocrinology 19, no. 6 (2005): 1569–1583, 10.1210/me.2004-0528. 15817653

[60] W. C. Huang and C. C. Chen , “Akt Phosphorylation of p300 at Ser‐1834 Is Essential for Its Histone Acetyltransferase and Transcriptional Activity,” Molecular and Cellular Biology 25, no. 15 (2005): 6592–6602, 10.1128/mcb.25.15.6592-6602.2005. 16024795 PMC1190347

[61] S. S. Koh , D. Chen , Y. H. Lee , and M. R. Stallcup , “Synergistic Enhancement of Nuclear Receptor Function by p160 Coactivators and Two Coactivators with Protein Methyltransferase Activities,” Journal of Biological Chemistry 276, no. 2 (2001): 1089–1098, 10.1074/jbc.M004228200. 11050077

[62] M. Winkle , S. M. El‐Daly , M. Fabbri , and G. A. Calin , “Noncoding RNA Therapeutics — Challenges and Potential Solutions,” Nature Reviews Drug Discovery 20, no. 8 (2021): 629–651, 10.1038/s41573-021-00219-z. 34145432 PMC8212082

[63] Z. Liu , Y. Fu , M. Yan , et al., “microRNAs in Kidney Diseases: Regulation, Therapeutics, and Biomarker Potential,” Pharmacology & Therapeutics 262 (2024): 108709, 10.1016/j.pharmthera.2024.108709. 39181246

[64] D. Mellis and A. Caporali , “MicroRNA‐Based Therapeutics in Cardiovascular Disease: Screening and Delivery to the Target,” Biochemical Society Transactions 46, no. 1 (2018): 11–21, 10.1042/bst20170037. 29196609

[65] A. A. Seyhan , “Trials and Tribulations of MicroRNA Therapeutics,” International Journal of Molecular Sciences 25, no. 3 (2024): 1469, 10.3390/ijms25031469. 38338746 PMC10855871

[66] J. A. Kellum , P. Romagnani , G. Ashuntantang , C. Ronco , A. Zarbock , and H. J. Anders , “Acute Kidney Injury,” Nature Reviews Disease Primers 7, no. 1 (2021): 52, 10.1038/s41572-021-00284-z. 34267223

[67] Z. Lu , Y. Guo , D. Xu , et al., “Developmental Toxicity and Programming Alterations of Multiple Organs in Offspring Induced by Medication during Pregnancy,” Acta Pharmaceutica Sinica B 13, no. 2 (2023): 460–477, 10.1016/j.apsb.2022.05.029. 36873163 PMC9978644

[68] L. A. Ortiz , A. Quan , A. Weinberg , and M. Baum , “Effect of Prenatal Dexamethasone on Rat Renal Development,” Kidney International 59, no. 5 (2001): 1663–1669, 10.1046/j.1523-1755.2001.0590051663.x. 11318936 PMC4127466

[69] J. R. Schelling , “Tubular Atrophy in the Pathogenesis of Chronic Kidney Disease Progression,” Pediatric Nephrology 31, no. 5 (2016): 693–706, 10.1007/s00467-015-3169-4. 26208584 PMC4726480

[70] J. Stenvang , A. Petri , M. Lindow , S. Obad , and S. Kauppinen , “Inhibition of microRNA Function by antimiR Oligonucleotides,” Silence 3, no. 1 (2012): 1, 10.1186/1758-907x-3-1. 22230293 PMC3306207

[71] A. D. Burdick , S. Sciabola , S. R. Mantena , et al., “Sequence Motifs Associated with Hepatotoxicity of Locked Nucleic Acid—Modified Antisense Oligonucleotides,” Nucleic Acids Research 42, no. 8 (2014): 4882–4891, 10.1093/nar/gku142. 24550163 PMC4005641

[72] S. Reagan‐Shaw , M. Nihal , and N. Ahmad , “Dose Translation from Animal to human Studies Revisited,” The FASEB Journal 22, no. 3 (2008): 659–661, 10.1096/fj.07-9574LSF. 17942826

[73] F. Närhi , S. R. Moonesinghe , S. D. Shenkin , et al., “Implementation of Corticosteroids in Treatment of COVID‐19 in the ISARIC WHO Clinical Characterisation Protocol UK: Prospective, Cohort Study,” The Lancet Digital Health 4, no. 4 (2022): 220, 10.1016/s2589-7500(22)00018-8. PMC8940185 35337642

[74] T. Dagklis , C. Sen , I. Tsakiridis , et al., “The Use of Antenatal Corticosteroids for Fetal Maturation: Clinical Practice Guideline by the WAPM‐World Association of Perinatal Medicine and the PMF‐Perinatal Medicine Foundation,” Journal of Perinatal Medicine 50, no. 4 (2022): 375, 10.1515/jpm-2022-0066. 35285217

[75] Y. Ao , Z. Sun , S. Hu , et al., “Low Functional Programming of Renal AT2R Mediates the Developmental Origin of Glomerulosclerosis in Adult Offspring Induced by Prenatal Caffeine Exposure,” Toxicology and Applied Pharmacology 287, no. 2 (2015): 128–138, 10.1016/j.taap.2015.05.007. 25986755

[76] B. Hohenstein , S. Renk , K. Lang , et al., “P2Y1 Gene Deficiency Protects from Renal Disease Progression and Capillary Rarefaction during Passive Crescentic Glomerulonephritis,” Journal of the American Society of Nephrology 18, no. 2 (2007): 494–505, 10.1681/asn.2006050439. 17215444

