# Supplementary material for: miR‐135a‐5p Is a Promising Target to Prevent the Glomerulosclerosis Associated with Podocyte Developmental Toxicity in Offspring Induced by Prenatal Dexamethasone Exposure
Source: Adv Sci (Weinh). 2026 Jan 30;13(20):e19743. doi: 10.1002/advs.202519743 (PMC13067830; doi:10.1002/advs.202519743)
Supplement: Supplementary file 1 — Supporting File: advs74128‐sup‐0001‐SuppMat.docx. [file ADVS-13-e19743-s001.docx]

Supporting Information

**miR-135a-5p is a promising target to prevent the glomerulosclerosis associated with podocyte developmental toxicity in offspring induced by prenatal dexamethasone exposure**

*Xiaoqi Zhao, Haiyun Chen, Yanan Zhu1, Zhiping Xia, Hangyuan He, Yutang Liu, Tianshu Yang, Hui Wang, Ying Ao**

**Supplementary date：**


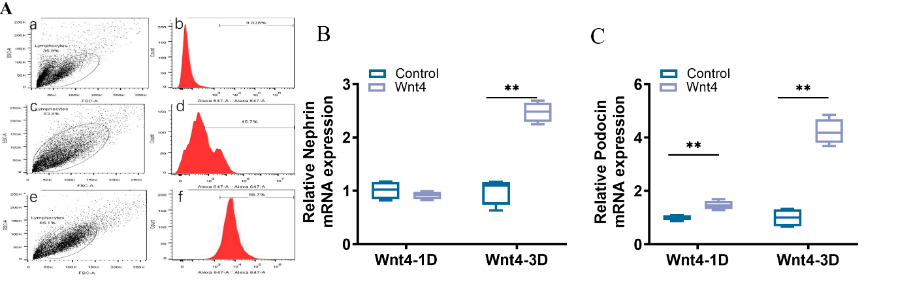


**Fig. S1. Establishment of a cellular model for the differentiation of MMSCs to podocytes.** A: Identification of Nephrin surface antigen by flow cytometry. a-b, MMSCs without Wnt4. c-d, MMSCs treat with Wnt4 (100 ng/mL) for 1 day. e-f, MMSCs treat with Wnt4 (100 ng/mL) for 3 days. B-C: Nephrin and podocin mRNA expression in MMSCs treated with Wnt4, *n* = 4.

. The *P* value was calculated using Student’s *t*-test, mean ± S.E.M. ^*^*P* < 0.05, ^**^*P* < 0.01 *vs* control.


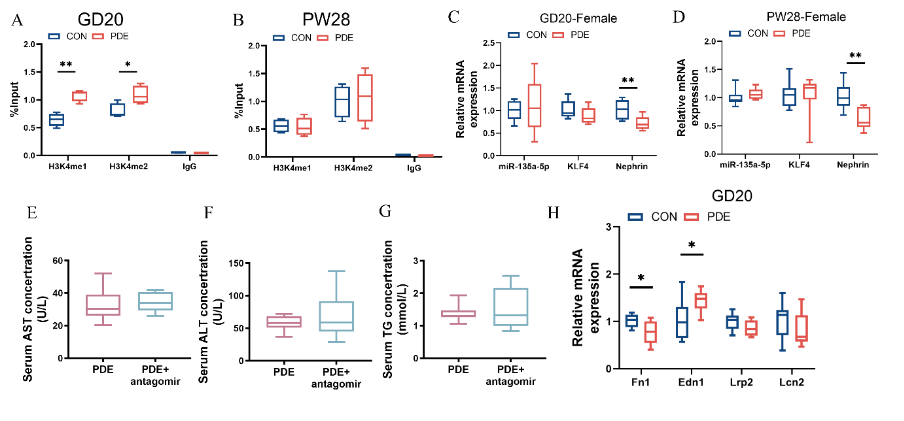


**Figure S2 A-B:** Enrichment of H3K4me1 and H3K4me2 in the miR-135a promoter was analyzed using ChIP-PCR in the rat offspring kidneys at GD20 and PW28, *n* = 4. **C-D**: mRNA expression of miR-135a-5p, KLF4 and Nephrin in the female rat offspring kidneys at GD20 and PW28, *n* = 6~10. **E-G**: The levels of AST, ALT and TG in serum of adult mouse offspring. *n* = 7~10. **H**: mRNA expression of Fn1, Edn1, Lrp2 and Lcn2 in the male rat offspring kidneys at GD20. *n* = 8~10. The *P* value was calculated using Student’s *t*-test or Mann–Whitney U test , mean ± S.E.M. **P* < 0.05, ***P* < 0.01 vs control. GD, gestational day; PW, postnatal week; CON, control; PDE, prenatal dexamethasone exposure; KLF4, Krüppel-like factor4; GAPDH, glyceraldehyde phosphate dehydrogenase; DEX: dexamethasone; AST, Aspartate Aminotransferase; ALT, alanine aminotransferase; TG, triglycerides.

**Table. S1. Oligonucleotide primers of rat in real-time quantitative PCR.**

| Genes | Forward primer (5′-3′) | Reverse primer (5′-3′) | Transcript (RefSeq / Ensembl) |
| --- | --- | --- | --- |
| **Rat:** |  |  |  |
| GR  (NR3C1) | CACCCATGACCCTGTCAGTG | AAAGCCTCCCTCTGCTAACC | NM_012576.2 |
| P300  (EP300) | CAAATGCAGGCATGGGCAAT | TCCTGGTTGTCCTCCCATCT | ENSRNOT00000115202 |
| KLF4 | GTGCCCCGACTAACCGTTG | GTCGTTGAACTCCTCGGTCT | NM_053713.1 |
| Nephrin | GAGAGGAGTCTAGGAAGATAGG | TTACTGGCAGCTTGGATTG | NM_022628.1 |
| Podocin | GTCTCCAGACCTTGGAAATAC | GGAACTGGATGGCTTTGG | ENSRNOG00000004030 |
| WT1 | GTACCCAGGCTGCAATAAG | CACCTGTGTGTCTCCTTTG | NM_031534.x |
| GAPDH | CTCCCATTCTTCCACCTTTG | TGGTCCAGGGTTTCTTACT | NM_017008.4 |
| **Mouse:** |  |  |  |
| KLF4 | CTATGCAGGCTGTGGCAAAACC | TTGCGGTAGTGCCTGGTCAGTT | NM_010637 |
| Nephrin | GCATAGCCAGAGGTGGAAATCC | GAACGGTCATCACCAGCACACT | NM_019459 |
| Podocin | GTGGAAGCTGAGGCACAAAGAC | CAGCGACTGAAGAGTGTGCAAG | NM_130456 |
| GAPDH | TGTGTCCGTCGTGGATCTGA | CCTGCTTCACCACCTTCTTGA | NM_008084 |

**Table. S2. siRNA sequences and miR-135a-5p antagomir used in this study.**

| Genes | Forward primer (5′-3′) | Reverse primer (5′-3′) | Transcript (RefSeq / Ensembl) |
| --- | --- | --- | --- |
| GR  (NR3C1) | GCAUGAAGAUCUCUGAAGA | UCUUCAGAGAUCUUCAUGC | NM_012576.2 |
| P300  (EP300) | GGAUGAAGCUCAACAAAUA | UAUUUGUUGAGCUUCAUCC | ENSRNOT00000115202 |
| miR-135a-5p antagomir | UAUGGCUUUUUAUUCCUAUGUGA |  | UAUGGCUUUUUAUUCCUAUGUGA |
